# Seasonal variation of a plant-pollinator network in the Brazilian Cerrado: Implications for community structure and robustness

**DOI:** 10.1371/journal.pone.0224997

**Published:** 2019-12-02

**Authors:** Simone Cappellari Rabeling, Jia Le Lim, Rosana Tidon, John L. Neff, Beryl B. Simpson, Samraat Pawar

**Affiliations:** 1 Department of Integrative Biology, The University of Texas at Austin, Texas, United States of America; 2 Department of Life Sciences, South Kensington Campus, Imperial College London, London, United Kingdom; 3 Departamento de Genética e Morfologia, Instituto de Ciências Biológicas, Universidade de Brasília, Brasília, DF, Brazil; 4 Central Texas Melittological Institute, Austin, TX, United States of America; 5 Department of Life Sciences, Silwood Park Campus, Imperial College London, Ascot, Berkshire, United Kingdom; Universidade de Sao Paulo Faculdade de Filosofia Ciencias e Letras de Ribeirao Preto, BRAZIL

## Abstract

Seasonal variation in the availability of floral hosts or pollinators is a key factor influencing diversity in plant-pollinator communities. In seasonally dry Neotropical habitats, where month-long periods of extreme drought are followed by a long rainy season, flowering is often synchronized with the beginning of precipitation, when environmental conditions are most beneficial for plant reproduction. In the Brazilian Cerrado, a seasonally dry ecosystem considered one of the world’s biodiversity hotspots for angiosperms, plants with shallow root systems flower predominantly during the rainy season. Foraging activity in social bees however, the major pollinators in this biome, is not restricted to any particular season because a constant supply of resources is necessary to sustain their perennial colonies. Despite the Cerrado’s importance as a center of plant diversity, the influence of its extreme cycles of drought and precipitation on the dynamics and stability of plant-pollinator communities is not well understood. We sampled plant-pollinator interactions of a Cerrado community weekly for one year and used network analyses to characterize intra-annual seasonal variation in community structure. We also compared seasonal differences in community robustness to species loss by simulating extinctions of plants and pollinators. We find that the community shrinks significantly in size during the dry season, becoming more vulnerable to disturbance due to the smaller pool of floral hosts available to pollinators during this period. Major changes in plant species composition but not in pollinators has led to high levels of turnover in plant-pollinator associations across seasons, indicated by in interaction dissimilarity (<3% of shared interactions). Aseasonal pollinators, which mainly include social bees and some solitary specialized bees, functioned as keystone species, maintaining robustness during periods of drastic changes in climatic conditions.

## Introduction

Some of the most impressive natural phenomena on Earth, such as the great migrations of wild ungulates in Africa, are linked to the effects of seasonal rainfall on trophic interactions between species (e.g., between primary producers and consumers) [1, 2]. While the influence of seasonal rainfall on plant growth and species-specific reproductive cycles has been widely documented in tropical habitats [3–5], its impact on multi-trophic interactions is less well understood. Yet, a large proportion of Neotropical habitats are characterized by cycles of drought lasting for several months followed by similarly long periods of intense rainfall [6]. Neotropical savannas are ecosystems characterized by this bi-seasonal climatic regime comprising the Cerrado, the Llanos, and some isolated, smaller pockets of vegetation scattered throughout South and Central America [6, 7]. Together these neotropical savannas originally covered a large proportion of the tropical Americas (estimated to be ~3 million km^2^) [7] and their conservation has been considered a global priority based on their high diversity and percentage of endemism [6, 8]. Despite their importance, these seasonal habitats are understudied and little information exists about the antagonistic effects of water stress and intense rainfall on species phenologies and subsequent temporal variation in plant-animal interactions at the community level [5, 9]. Species interactions, especially with pollinators, along with climatic factors have long been recognized among major biotic and abiotic factors promoting neotropical plant diversification [10–14]. Understanding how plant-pollinator communities from such habitats are organized and how they function under such contrasting climatic conditions is important not only for gaining insights about factors promoting the high diversity characteristic of Neotropical ecosystems, but also for estimating community responses to future changes in climate [5].

The Brazilian Cerrado ranks as one of the most biologically diverse seasonally dry ecosystems in the Neotropic region, comprising about 10% of its plant diversity, including a large proportion of endemic species [15, 16]. In addition to a highly diverse flora and entomofauna in general, the Cerrado is considered a center of Neotropical bee diversity [17–19]. Pollination by bees is the predominant mode of pollen transfer among Cerrado plants [20–24]. Over the past few decades, major progress has been made in characterizing seasonal patterns of insect abundance [25–29] and the relationship between plant phenology and seasonality in this ecosystem [3, 20, 22, 23, 30, 31]. However, the extent of seasonal or intra-annual variation in the distribution and frequency of plant-pollinator interactions in Cerrado communities remains largely unexplored.

Empirical studies have shown that a large number of Cerrado plants, especially herbs and shrubs, flower primarily during the rainy season months, while woody species can flower in both seasons or mainly during the dry season [3, 16, 30, 32–34]. Such differences in phenological strategies have been in part attributed to the inability of shallow rooted herbs and subshrubs to utilize water stored in deeper soil layers during the dry season, which remains available to woody species and is necessary to support energy costs associated with the production of fruits [33–37]. In contrast to plants, rainfall and drought have not been shown to pose equally strong constrains on the foraging activity of pollinators in this habitat, primarily because a large percentage of Cerrado bees are social with perennial colonies active throughout the year [17, 18, 38].

Intra-annual variation in community composition and in the distribution of plant-pollinator interactions are expected in tropical and temperate communities. Such variation can result from several factors, including differences in timing and length in the phenology of plants and activity of pollinators and population dynamics [39]. Two factors appear to play an important role promoting robustness in communities subject to high temporal variation in species composition: interaction redundancy and rewiring. Interaction redundancy is defined as the interchangeability between species that perform ecologically similar including pollinator or floral hosts within a plant-pollinator community [40, 41]. Thus, a community with high levels of redundancy would become more robust as different species are able to “replace” each other if fluctuations in natural population lead to lower rates of interaction between particular pairs of species [40, 42]. In addition, rewiring—the ability of many plant and pollinator species to form new associations with mutualistic partners—has been increasingly considered to be another key factor buffering temporal species fluctuations in natural communities [42, 43]. Because both factors rely on diversity of mutualistic partners to operate, it is possible that a seasonal constriction in the species pool could render communities more vulnerable to disturbance.

Here, we investigated and compared temporal variation in the topology and robustness of a Cerrado plant-pollinator community during its rainy and dry seasons. Specifically, we asked: (a) whether the plant-pollinator community differs in key structural features between seasons; (b) which biological factors explain community variation through time; and (c) whether temporal changes in structure can reduce the community’s robustness to species loss in either season. Because our study site comprised mainly herbs and shrubs with few scattered trees, i.e., a Cerrado-typical phytophysiognomy known as “campo sujo,” we predicted that a lower diversity of floral hosts should be expected in this community during the dry season. Using ecological network tools and statistical analyses of community dissimilarity, we characterized and compared this community across seasons to determine its structural variation over time. Finally, we evaluated seasonal differences in community robustness to species loss by simulating and comparing the effect of species extinctions in each season.

## Methods

### Study area and climate information

**Fig 1:**
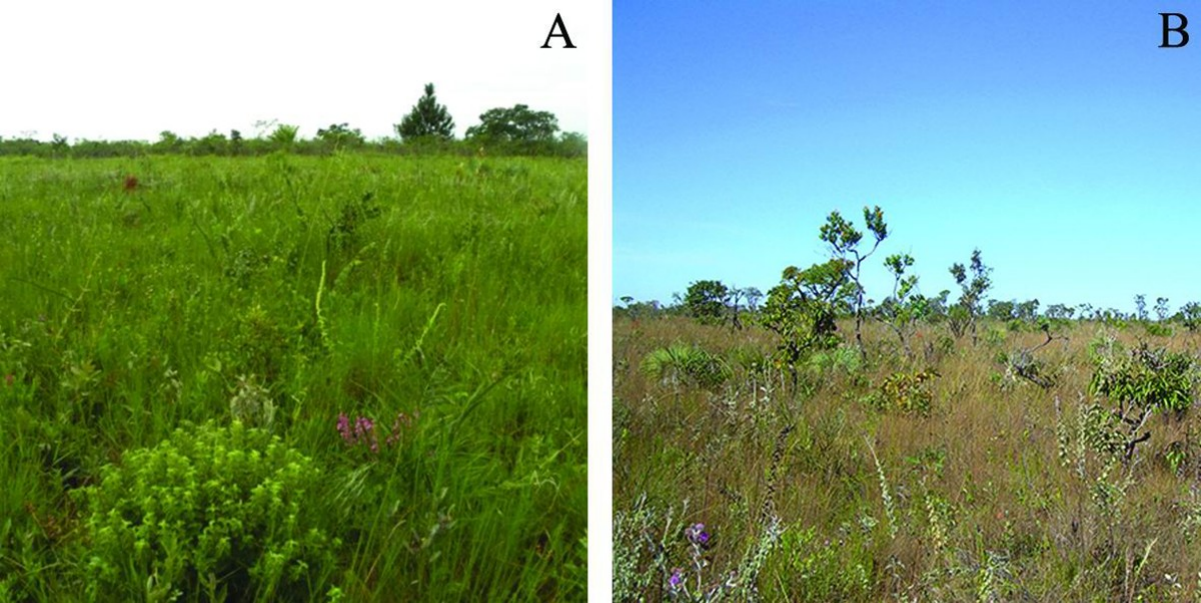
Overview of study site. Images illustrate the vegetation during the rainy (left) and dry (right) seasons at the Reserva Ecológica do IBGE, Brasília, Brazil (Photographs by S. C. Rabeling).

Field work was carried at the protected nature reserve, Reserva Ecológica do IBGE (RECOR-IBGE), with permission of IBGE’s employer and RECOR’s director Mauro Lambert Ribeiro. Located 30 km south of the capital Brasília at 1,100 m ASL, this nature reserve is part of a larger, federally protected conservation area known as “APA-Gama-Cabeça-de-Viado” (15° 56’ S and 47° 53’ W). This region is characterized by a well-defined dry winter season that lasts from May until September followed by a summer rainfall period extending from October until March or April [30]. The study area comprised a 8-hectare plot (200 x 400 m) covered with the phytophysiognomy known as *campo sujo* (Fig 1), a relatively open type of vegetation mainly composed of grasses mixed with herbaceous plants, and shrubs, with sparse occurrence of lianas and trees [16, 30]. This area was sampled weekly by one person walking parallel, adjacent transects sampling the entire area during a full day (0800h to 1700h). Data collection was performed from October 2008 to September 2009. The sampling effort totaled 47 sampling days over a 12-month period.

We used data from the IBGE’s weather station to evaluate and compare monthly average temperatures and precipitation for the year of our study (2008–2009) and those recorded in the past 30 years (1980–2010) (S1 Fig). According to these data, 88% of total annual precipitation (mean monthly precipitation = 6.82 cm; s.d. = 3.22 cm) occurs between October and March, with average temperatures ranging from 25.7°C (s.d. = 1.41° C) during the day to 19.2° C (s.d. = 1.3°C) at night. Mean monthly precipitation drops to 1.15 cm (s.d. = 1.6 cm) in the remaining period with average temperatures during the day at 24.3°C (s.d. = 1.83° C) to 18° C (s.d. = 1.9° C) at night (S1 Fig). As already pointed by other authors [16, 30], the months of April and October present variable climatic conditions reflecting the transition between the seasons (S1 Fig). For analytical purposes, we assigned the interactions recorded in the transitional months of April and October to the dry and rainy seasons, respectively. The periods defined for each season agree with patterns reported for this and other nearby Cerrado areas [15, 20, 30, 44].

### Sampling methods and species identification

Empirical studies have shown that bees are the predominant pollinators in Cerrado communities (~ 70%) followed by moth pollination (~ 12%) and a smaller percentage of hummingbirds (~ 3%), bats (~ 2%), and beetles (~ 2%) [20, 21, 23, 31, 38]. In view of their overwhelming importance as pollinators, we chose to focus on and record interactions involving bees only disregarding visits involving other types of pollinators such as hummingbirds, flies, etc., as a representative data set for this highly diverse tropical habitat. Because our study aimed at detecting major structural changes in plant-pollinator communities during dry and rainy seasons, we chose to use a weekly sampling approach to obtain a comprehensive data set and capture small changes in climatic conditions as well as plant and insect phenology. Each week, bees were collected with an insect net and killed either in individual vials with paper pellets moistened with ethyl acetate or frozen after each observation. A plant or pollinator species observed in the study plot was considered part of the community and included in each of the surveys only if it were involved in an interaction. For every new interaction recorded, a plant was tagged with a unique identification number, photographed, and vouchered. Photos of each plant species were added to a database of local floral hosts, and a secondary miniature voucher was made to facilitate re-identification of species in the field. Plant vouchers were identified by using comparative herbarium material, a checklist of local angiosperms, and local botanical expertise (see acknowledgments section). Vouchers of the plants sampled were deposited at the herbaria of the IBGE research station (IBGE) and the University of Brasília (UB). Insect vouchers were mounted, preserved, and identified to species level by comparison with reference collections, the taxonomic literature, local records [19, 45–48], and by local entomological experts (see acknowledgments). All insect vouchers were deposited in the entomological collections of the University of Brasília and the Padre Moure Collection at the Federal University of Paraná, Brazil.

### Data analyses

We used a combination of ecological network, β-dissimilarity, and correlation analyses to identify temporal changes in community structure at our study site. The sampling effort was evaluated through a rarefaction analyses considering each season and the number of new unique interactions recorded (i.e., new species links) as sampling units. We used the number of links sampled instead of days in this analysis to prevent the confounding effects associated with variation in the number of sampling hours per day due to of inclement weather conditions that occasionally interrupted field work during the rainy season. The sequence of links recorded was randomized a hundred times before accumulation curves for network features were generated. We calculated 95% Confidence Intervals (CIs) around each curve to determine the overlap between the observed and the expected curves for each network feature with regard to the number of new links sampled. All analyses and simulations were performed using code written in Octave, Python, and R.

#### a. Seasonal variation in community parameters

To test whether the community shrank in size during the dry season, and to investigate which factors contributed to such potential size reduction, we measured and compared specific community attributes for both seasons over time. First, we calculated the total number of links (i.e., interactions recorded between plant-bee species pairs, *L*) and interaction frequency (*F*) calculated as the number of visits to *i*^th^ plant by *j*^th^ pollinator relative to the number of flowering instances recorded for that plant species (by counting all instances of that plant having been visited by any bee) for all species in the network. These measures were then used to calculate the following network measures:

- *Network size*; simply the number of nodes (total number of species observed in our study) and was calculated for each season and for the entire year.
- *Bipartite connectance;* the proportion of interactions or links that are realized (observed) divided over the total possible links between the two trophic levels (i.e., bees and plants; BC = 2*L* / (*N*_po_ *N*_pl_)). Connectance and diversity of mutualistic networks have been shown to be positively correlated [49]. Thus, we expected network connectance to drop in the dry season because a lower diversity of floral host species was expected during this period.
- *Species’ degree*; the number of partners for each species (i.e., specialization level). Because the lower diversity of available species, especially of floral hosts, can reduce the number of potential partners available during the dry season, narrower niche widths were expected for species active this season. *Degree distributions* were also compared between seasons using three alternative models (exponential, power-law, and truncated power-law), with the Akaike Information Criteria (AIC) to evaluate each fit.
- *Weighted species’ degree*; the sum of interaction frequencies across all partners of a species. The interaction frequency between a bee-plant pair was calculated by dividing the number of visits by the bee to the plant species by the number of days that plant was observed interacting (with any bee) across the sampling period (in each season). That is, all instances of that plant having been visited by any bee were used as a proxy of its flowering activity. This approach reduces underestimation of interaction frequencies by accounting for plants not flowering during the entire period in which pollinators were active. Weighted degrees also quantified the relevance of different interaction types at different times of the year [50].
- *Nestedness*, a measure calculated using the Node Overlap and Decreasing Fill (NODF) measure [51]. Nested networks have been associated with increased robustness [52–54] and this measure was calculated to determine if such pattern would explain the distribution of species and their links across seasons.

#### b. Seasonal variation in plant-pollinator interactions

For evaluating and comparing the level of temporal variation in the studied community during the two Cerrado seasons, we calculated *β*-dissimilarity measures and analyzed their relationships. We calculated monthly interaction turnover for all species in the community (*β*_int_) using Whittaker’s presence-based dissimilarity measure [55]:

$$\beta int=\frac{a+b+c}{(2a+b+c)/2}-1$$ Eq 1

Here, *a* is the number of interactions present in two successive monthly networks while *b* and *c* are the number of unique interactions present in each of the networks respectively [56]. Thus, *β*_int_ quantifies the differences, or dissimilarity, of interactions between each pair of monthly networks. The measure is 0 when the interaction networks of two subsequent months are identical and 1 when the two interaction networks do not have any elements in common [41, 56]. Whittaker’s index is more robust than other *β*-dissimilarity indices when dealing with heterogeneous dataset sizes [56, 57]. Furthermore, *β*_int_ can be partitioned into two components: network dissimilarity due to species turnover (*β*_st_) and interaction rewiring between shared species of networks (*β*_rw_):

βint=βst+βrw Eq 2

In theory, *β*_st_’s contribution to *β*_int_ covaries with species turnover (*β*_s_), the difference in species composition of seasonal networks [56]. The measures *β*_rw_, *β*_st_, *β*_po_ and *β*_pl_ were themselves also calculated using Eq 1, where *a* now refers to the number of interactions between shared species or species present in the community in two consecutive months; and *b* and *c* refer to the number of unique elements present in each of the two months. These elements refer to interactions between species common to networks in both months, species, bee species, and plant species for the measures *β*_rw_, *β*_st_, *β*_po_ and *β*_pl_, respectively (S1 Table). For this analysis, *β*_st_ was obtained by subtracting *β*_rw_ from *β*_int_ (Eq 2). Overall, these analyses enabled us to determine whether the seasonal differences in networks were driven by a high turnover in plant species, pollinator species, or both, and to what extent seasonal changes in plant-pollinator interactions arise due to rewiring (i.e., establishment of new types of interactions).

We then performed correlation analyses to investigate the relationships between the different dissimilarity measures to determine whether the level of dissimilarity observed is driven by similar underlying factors. For example, a potential correlation of interaction turnover with plant or pollinator species turnover would indicate that changes in interactions are mainly driven by changes in species at a particular trophic level. As turnover measures are not normally distributed, we calculated the non-parametric Spearman’s rank correlation coefficient, *r*_s_ [58]. Furthermore, network turnover between timepoints is non-independent because if, for instance, the interaction network of April was found to be altered, this would have affected both March-April and April-May plant and interaction turnover rates, resulting in non-independence of turnover measures and rendering the *p*-values of the Spearman’s test biased. To overcome this, a Monte Carlo procedure was used to generate the *p*-values of the correlation tests obtained from the data. Specifically, randomized sets of bees and plants were drawn across the dataset to form 10^5^ simulated networks. Correlation coefficients between turnover measures were calculated for each simulation generating a sampling (null) distribution of *r*_s_ values between turnover measures of interaction networks resulting from random sampling and rewiring. The numbers of bees, plants and interactions, as well as connectance of each simulated monthly network were kept constant. P-values were then obtained by comparing the observed the seasonal interaction turnover rate to the null distribution.

#### c. Seasonal variation in network robustness

We performed species extinction simulations to study how seasonal variation in network structure affected the community’s robustness to loss of functionally important species. In particular, a lack of interaction partners in a seasonally reduced (i.e., with fewer species) community could influence its ability to recover and maintain function in the face of perturbation through species loss or decrease in abundances due to factors such as habitat destruction. For this, we used information about community composition as well as niche breadth, represented by weighted and unweighted degrees, to guide targeted species removal. Unweighted robustness analyses followed established methodology [59–61]: upon removal of a plant or pollinator node, every pollinator or plant species left without interaction partners was considered extinct. For the weighted robustness analyses, we considered targeted removal of species according to their importance in terms of connections maintained in each seasonal period as well as the overall network (i.e., from widest to narrowest niche breadth in order of decreasing degree). In addition, we also considered scenarios of targeted removal from weakly to highly connected species (i.e., in order of increasing niche breadth), as well as at random.

Weighted robustness was calculated following a procedure similar to Kaiser-Bunbury et al. [62]. We measured proportional loss of visitation rates, using the link weights defined above, instead of just species loss. For this, we first converted each link weight to its fractional contribution to the total visitation rates across the entire network. All robustness simulations of plant and pollinator removals were performed individually. When multiple nodes with the same niche width values (i.e., degree) were encountered in targeted removal sequences we simply permuted the removal sequence among them to break ties. Thus, random as well as targeted removals yielded a sample of extinction curves. The values at each point on this curve were then averaged to obtain a single curve. For both weighted and unweighted robustness analyses, the Area Under the Curve (AUC) was used as a measure of network robustness to species loss. We chose 100 iterations as a compromise between computational complexity and accuracy of the average extinction curve. To estimate whether extinction curves were significantly different, we calculated 95% confidence intervals (CI’s) around each mean value on the curve. We also calculated number of dependent species lost after loss of 50% of target species (also with 95% CI’s).

## Results

### Seasonal variation in community structure

**Fig 2:**
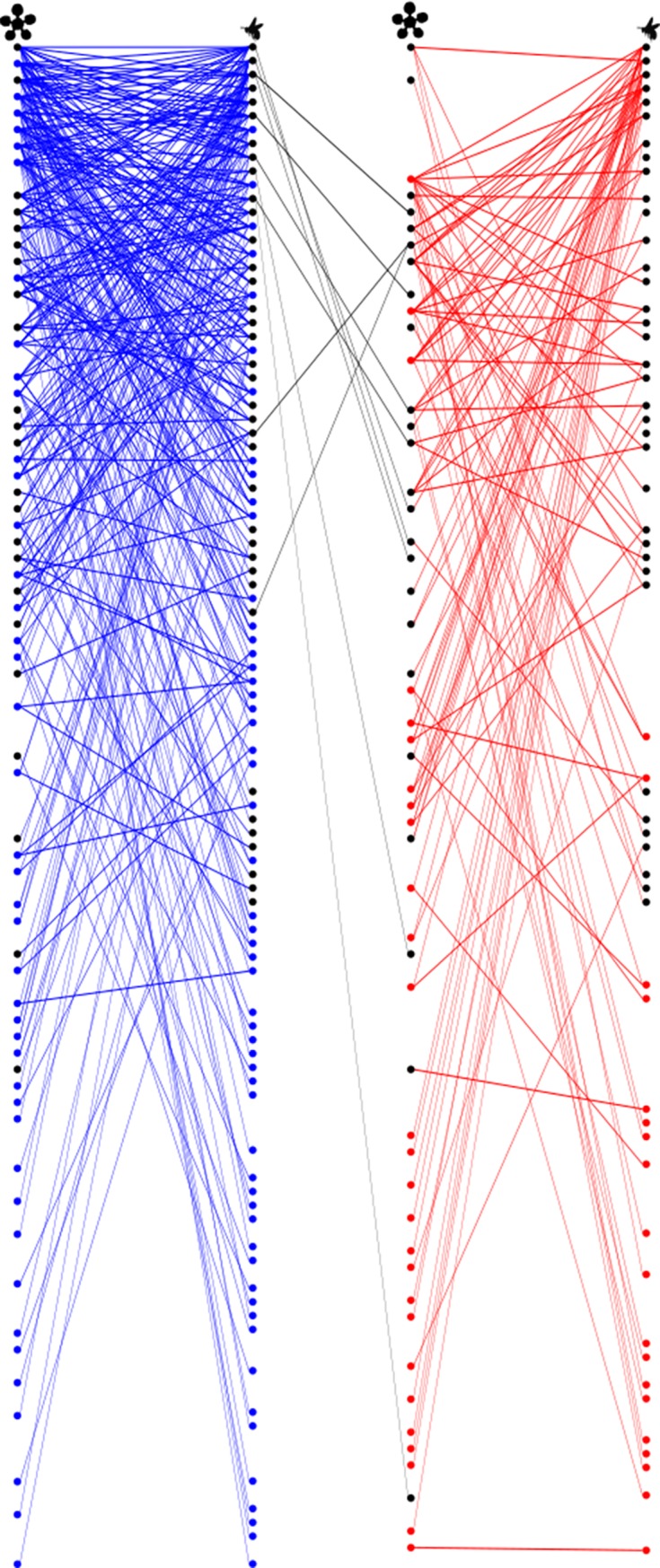
Plant-pollinator network of the studied Cerrado community, color-coded by season. Both plants (left, flower symbol) and animal (right) nodes have been ordered by decreasing degree (i.e., by number of interacting species) from top to bottom. Blue nodes and links correspond to interactions recorded exclusively during the rainy season, red links to those recorded during the dry season only, while black links represent interactions overlapping between seasons. Each node is also color-coded to reflect species’ phenology: blue–rainy season only; red–dry season only; black–both seasons. Black nodes are clustered towards the top, indicating that generalized species are also typically found in both seasons. There is little interaction overlap (note the paucity of black links) between seasons, resulting in temporally distinct networks in both communities due to inter-seasonal turnover in floral hosts.

Over the 12-month study period, 93 species of plants and 111 species of flower foraging bees were recorded in IBGE. A total of 968 pollinator visits to plants, which constituted 434 unique associations (i.e., links) between plants and pollinators, were observed (Fig 2). Eleven species of semi- or eusocial bees were collected, most of which belonged to the native stingless bees tribe (Meliponini). However, the majority of species recorded were solitary bees and 28% of all bee species recorded belong to taxonomic groups (i.e., Centridini, Tapinotaspidini, and Tetrapediini) known to collect floral oils (n = 32) [19, 48].

**Fig 3:**
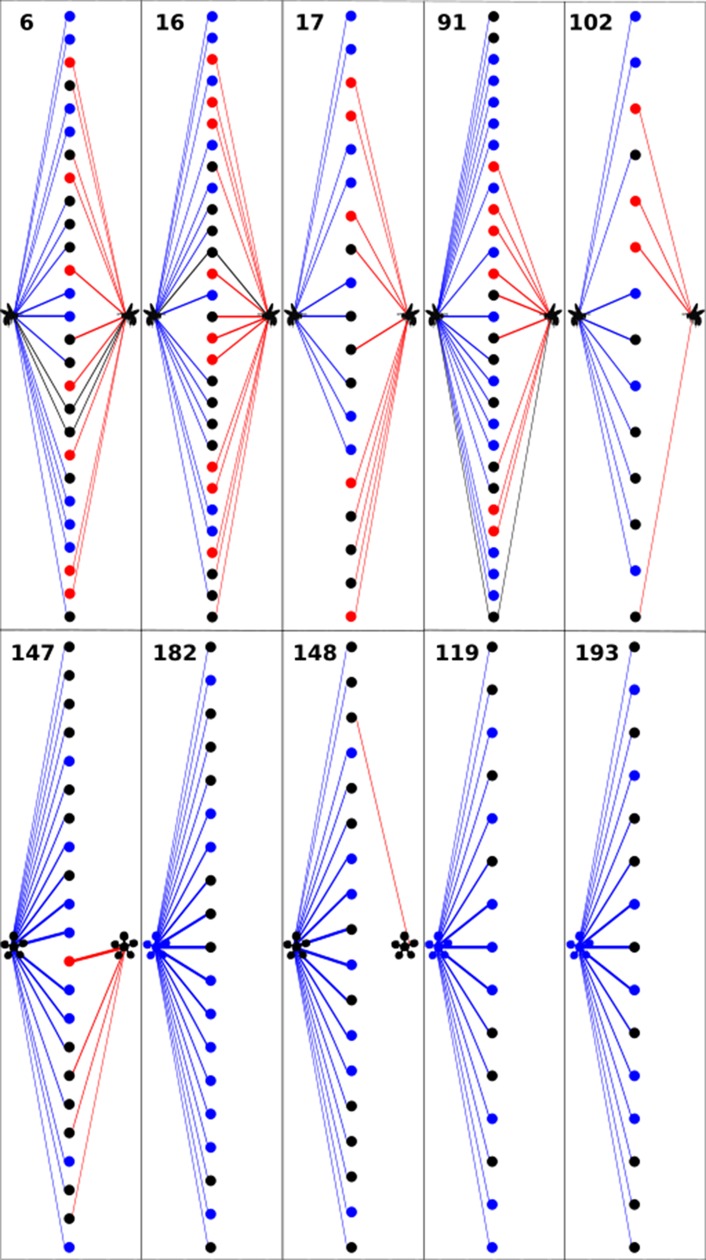
**Temporal turnover in the number of interaction partners for pollinators (upper panels) and plants (lower panels) in the studied Cerrado community.** Turnover is shown for the five species with the largest number of interaction partners in each community (i.e., top pollinators and plants are most linked). Numbers indicate species node identification number in the community (see S2 Table in the supplemental information for corresponding species names). Aseasonality of pollinators is illustrated by links colored in black. All top five pollinators were species of social bees (i.e., honey bees, stingless bees, and bumble bees). Seasonal transitions are associated with nearly complete turnover in the set of partner species with which plants and pollinators interacted. Each node and interaction is color-coded to reflect seasonal period of activity: blue–rainy season only; red–dry season only; and black–both seasons.

The top ten pollinator species with the widest niches (i.e., that interacted with a large number of species or had a degree > 8) in this community maintained a large number of interaction partners in both seasons (Fig 3; S2 Table). The majority of these abundant, well-connected, and *aseasonal* species represented mainly social bee species. About one third of all interactions recorded involved social bees, with the introduced honey bee (*Apis mellifera*) being the most abundant species (n = 154) sampled on flowers followed by species of the native stingless and bumble bees (S3 Table). Even though solitary bees were less abundant (i.e., interacted with less frequency) than social species at our study site, some solitary species were highly connected (i.e., had a high degree or number of interaction partners) to plants at this community (S2 Table). Among solitary bees, species of oil-collecting bees (i.e., tribes Centridini, Tapinotaspidini, and Tetrapediini) had a relatively large number of interactions and together comprised one third of all bee species (32 spp.) and 17% of all visits recorded at this site (S3 Table). The pollinator community composition in IBGE was similar to those observed in other Cerrado areas [17, 18] with Apidae being the richest group (77 spp.) followed by Halictidae (19 spp.), Megachilidae (13 spp.), Andrenidae (1 sp.), and Colletidae (1 sp.).

Plant species recorded flowering and receiving pollinator visits were mostly herbs and shrubs, representing 24 angiosperm families. Fabaceae (18 spp.) was the most species rich plant group involved in pollinator interactions (S2 Table). Among plants, *Dimerostemma vestitum* (Asteraceae; herb; degree = 22), *Palicourea coriacea* (Rubiaceae; shrub; degree = 19), and *Diplusodon oblongus* (Lythraceae; herb; degree = 18), which flowered mainly during the rainy season, displayed the widest niches, i.e., had the largest number of visiting pollinator species, and consequently the highest degrees (i.e., highest number of interaction partners) in cumulative accounts of species interactions (Figs 2 and 3). These species were major nectar and pollen sources for visitors. Unlike the most connected pollinators, plant species that had the largest number of interaction partners (i.e., high degrees) and received the largest number of visits (i.e., high visitation frequency) usually bloomed only in one season (S3 Table).

Our analyses of the sampling effort (S3 and S4 Figs) show that an asymptote was reached for the curve of pollinators’ mean number of interaction partners. No asymptote was reached for all the other parameters in the study site indicating that additional sampling efforts, i.e., bi or tri-weekly, would be necessary for complete accounts of the richness and diversity of these communities and their structural features.

### Biological factors underlying seasonal variation in Cerrado’s plant-pollinator interactions

The community was characterized by a strong and significant seasonal segregation formed of subsets of interacting species, with a remarkably small number of links shared between the rainy and dry seasons (Fig 2). The overlap in interactions (i.e., links) between seasons was 2.5% (11/434) and seasonal interaction clusters arose from significant and substantial turnover rates in species composition (*p* < 0.001; Table 1; Fig 2). There was a 74% turnover in plant species flowering and 67% turnover in pollinator species foraging between seasons. During the dry season, the number of flowering plant and foraging bee species was reduced, and only one-third of the total interactions recorded at the study site was observed during this period. In addition, network size, bipartite connectance, and mean species degree were all significantly lower during the dry versus the rainy season (Table 1). Degree distributions of both plants and pollinators followed a truncated power-law, indicating that species with narrow niches and few interaction partners (≤ 5 links) made up the bulk of species in this community in both seasons (S4 Table). Few species maintained a large number of partners (S2 Table) across seasons.

**Table 1:** Seasonal changes in network structure and species niche breath in a plant-pollinator community from the Cerrado.

Dimension	Measure	Cumulative	Rainy	Dry
Network structure	Network size (total species count)	204	158*	107*
	Plant species (*N*_pl_)	93	66	51
	Pollinator species (*N*_po_)	111	92*	56*
	Number of links (*L*)	434	318*	127*
	Number of visits recorded	968	608	360
	Bipartite connectance	0.084	0.105*	0.089*
	Nestedness (NODF)	13.9	13.3	10.2
Niche breadth	Mean plant degree	4.7	4.8*	2.5*
	Min plant degree	1	1	1
	Max plant degree	22	19	12
	Mean pollinator degree	3.9	3.5*	2.3*
	Min plant degree	1	1	1
	Max pollinator degree	29	20	14

Significant differences in measures between dry and rainy seasons (at one-tailed p < 0.05) are flagged with a “*” (see Methods). Each nestedness value is associated with a test of the null hypotheses that the observed network is no more nested than a random one (p < 0.0001), calculated from constrained network randomizations (see Methods).

**Fig 4:**
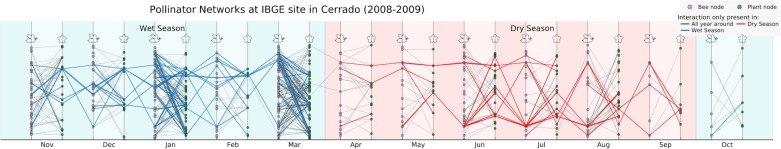
Turnover in monthly bee pollinator networks from Oct 2008 to Sep 2009. Every node in a monthly network represents a distinct plant or bee species. Each link between plant and bee nodes represent a unique pollination visit in the corresponding month. Links between monthly networks show interactions recorded in both monthly networks. Background of plot is color-coded to reflect the seasons: blue–rainy season; red—dry season. Lighter shaded areas demarcate the pollinator networks of each month, while darker areas demarcate the links present between monthly networks.

**Fig 5:**
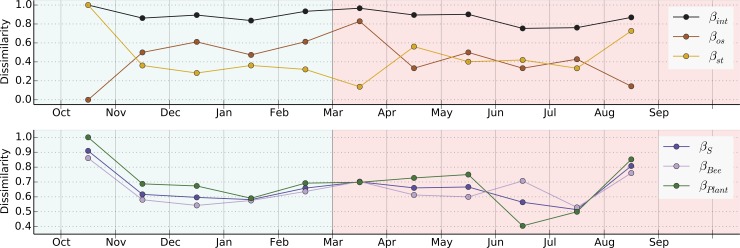
Correlation results for plant turnover (*β*_pl_) and interaction turnover (*β*_int_) rates in the studied community. A high *β*_pl_ and/or *β*_int_ value indicates a high dissimilarity between interactions and/or plant composition (r_s_: *Spearman*’s rank correlation coefficient; *p*: *p*-values generated using *Monte Carlo* simulations.).

Our analyses indicate that 4.83% of all unique interactions appeared in three or more monthly networks of the community (Fig 4). The monthly rate of interaction turnover (*β*_*int*_) was consistently high, ranging from 0.747 to 1 (S5 Table; S4 Fig). Interaction turnover (*β*_*int*_) was positively correlated with species turnover (*β*_*s*_) between months (Spearman’s coefficient, *r*_*s*_ = 0.809, *p* = 0.012) (S6 Table; S5 Fig). In particular, our analysis indicates a positive correlation between interaction turnover (*β*_*int*_) and plant species turnover (*β*_pl_) (*r*_*s*_ *=* 0.773, p = 0.012; S6 Table; Fig 5), which did not occur by chance, i.e., was not the product of random rewiring or random formation of new types of association between plants and pollinators.

Moreover, there is a relatively weak and non-significant correlation between interaction rewiring (i.e., new types of plant-pollinator associations, *β*_rw_), and general species turnover (*β*_S_) (r_s_ = -0.164, *p* = 0.359) (S5 Fig), indicating that factors driving interaction changes are different from those driving species turnover. Although there is a high correlation value between pollinator species turnover (*β*_po_) and plant species turnover (*β*_pl_) this relationship was not significant (*r*_*s*_ = 0.582, *p* = 0.116; S6 Table). Species turnover (*β*_S_) has a strong and non-random positive correlation with plant species turnover (*β*_pl_) (r_s_ = 0.964, *p* = 0.002; S6 Table), suggesting that the main driver of species turnover at this community was due to changes in flowering by different plant species across the seasons.

### Influence of seasonal changes in composition and structure for community’s robustness to species loss

**Fig 6:**
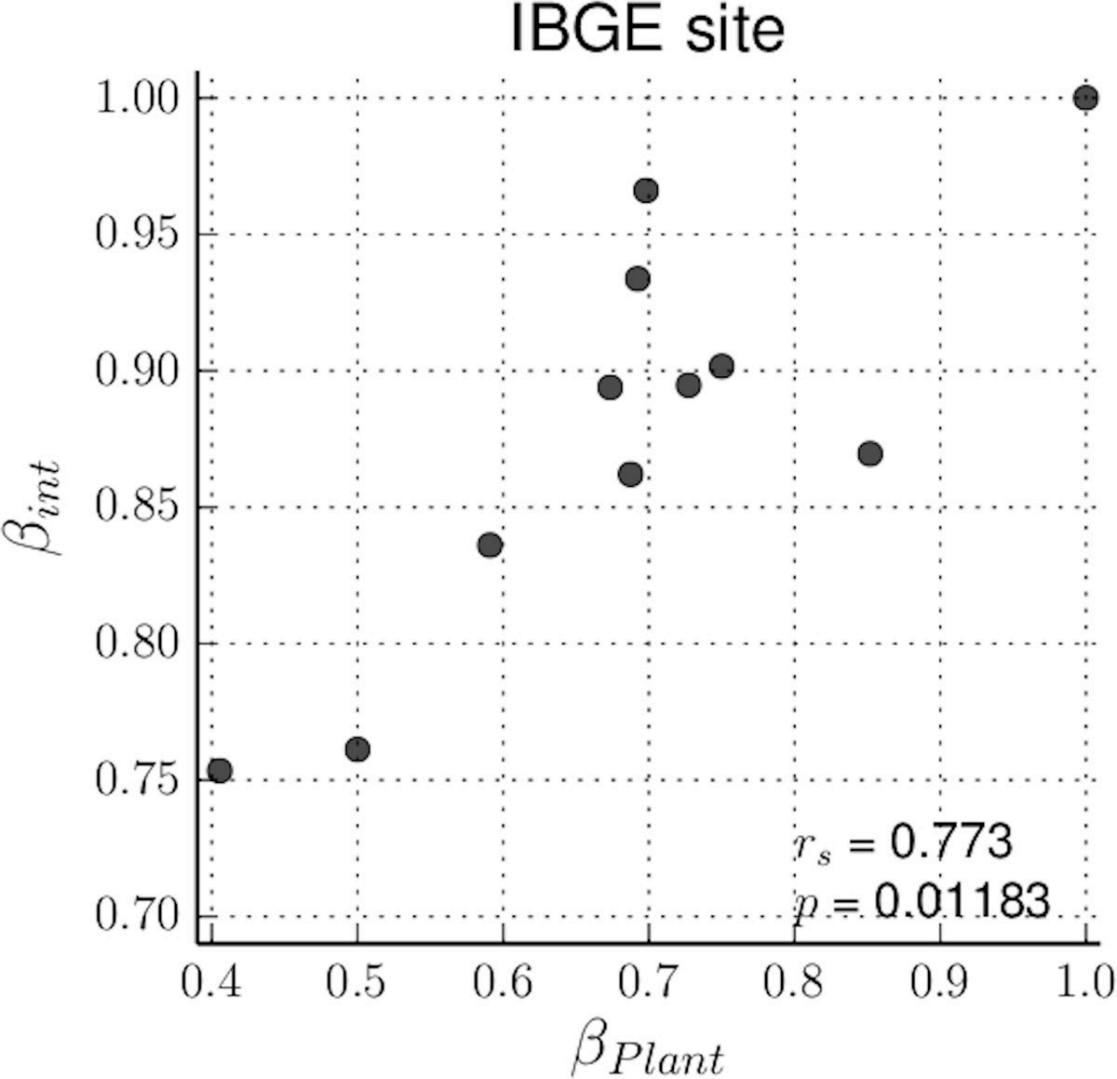
Network robustness to the simulated extinctions of pollinator and plant species in the studied community. Curves reflect extinction simulations results with removal following a decreasing rank of species importance (i.e., from largest to smallest values) in degree for robustness to species loss, and weighted degree for robustness to loss of flow. Dots indicate species loss robustness results as the proportion of species remaining in the community after removal; lines indicate flow robustness as the proportion of visitations being made or received after removal; red indicates dry season results, blue refers to interactions recorded during the rainy season, and black refers to the results considering the entire, aseasonal community. Significance of differences in robustness are indicates by asterisks for the removals by decreasing aseasonality (degree) in terms of overlap in the 95% confidence intervals (CI) around the AUC curves, as well as the proportion of nodes or visitation services remaining after removal (in parentheses, ±95% CI). For significance of differences in robustness of low to high degree and random removal of species, see supplementary materials.

Our simulations show that the community studied was less robust to pollinator removal relative to plant removal (S7 Table; Fig 6). This is consistent with our result that seasonal subsets of the community are coupled by bees, which maintain activity throughout the year, whereas plant flowering was mostly seasonal (S2 and S3 Tables; Figs 2 and 3). Moreover, robustness was lower when most-connected pollinators (i.e., the *aseasonal* species with high degrees) were removed first (S7 Table; Fig 6). Overall, the community was less robust to loss of visitation services (weighted robustness) than simple species loss (unweighted robustness). Furthermore, the subset of the community interacting during rainy season was the more robust to both species loss and visitation loss than the subset of species interacting during the dry season (S7 Table; Fig 6), an outcome consistent with the decrease in species degree (generality) for plants and pollinators. This result is also consistent with lower connectance observed in the community during the dry season (Table 1). Overall, similar changes in robustness were observed with season or aggregation for random species removal or targeted removal in order of increasing degree (S7 Table).

## Discussion

Our study focused on the characterization of seasonal variation in plant-pollinator interactions, community structure, and robustness in the Cerrado, a highly diverse tropical, seasonally dry, Neotropical ecosystem. Our analyses show that the plant-pollinator network of this community undergoes substantial structural changes between seasons, becoming not only smaller but also more vulnerable to disturbance during the dry season. Plant phenology was highly seasonal leading to high turnover rates in plant species composition between seasons in the community studied. Among seasonal differences observed in this network was low connectance, which is associated with a high proportion of species with narrow niches (i.e., ecological specialists). Such species are vulnerable to species extinctions or population fluctuations because they rely on few other partner species to for floral resources or pollination services [59, 63]. Conversely, communities comprising a large proportion of generalists are considered to be relatively stable because redundancy in partner associations is expected to buffer oscillations of mutualistic partners [40, 42]. Our network analyses also show that most interactions in this community occurred within a single season—less than 3% of all species interactions happen in both. Such low overlap in interactions between seasons can be explained by a combination of high turnover in the composition of flowering plant species with a moderate to low turnover in active pollinator species in this community. About 25% of the pollinators recorded over the study period interacted with a relatively high number of floral hosts in both seasons. When this community is analyzed as an interaction network, some of these pollinators function as *keystone species* because they visit and connect seasonal plant species (i.e., with phenologies restricted to either season) with the rest of species of this interaction network. As demonstrated for other Neotropical communities, several of these ‘aseasonal keystone pollinators’ were social or semi-social bee species, mostly stingless bees, which live in year-round active colonies producing multiple generations of individuals per year. The foraging period of workers in these colonies extend beyond the flowering periods of most individual plant taxa [64].

While social bees are known to be major pollinators of Neotropical plant communities due to their generalists foraging behavior [65], specialized solitary bees are not usually considered to have this role. However, in our study site, some species from solitary Neotropical oil-collecting bee groups (Apidae tribes Centridini, Tapinotaspidini, and Tetrapediini) also functioned as keystone species of the community. Despite being mostly univoltine (i.e., produce one generation per year), female oil-collecting bees can behave as generalists when harvesting the various resources necessary for their own feeding, nest construction, and larval provisions [66]. Oil-collecting bees, like most bees, use nectar for their own feeding and pollen as provision for their larvae [66]. However, these bees are unique in their use of floral oils (i.e., lipids) for nest construction and as an additive, mixed with pollen, in larval provisions [64, 66, 67]. Because most oil producing plants in the Cerrado do not secrete nectar [38, 68], oil-collecting bees need to visit multiple plant species to obtain all resources they need to survive and reproduce [66, 67]. The relevance of aseasonal pollinators, as observed in our Cerrado site, for the robustness and dynamics of the communities analyzed here is likely to be common to many, if not most Neotropical systems, where warm and stable climates can promote plant-pollinator interactions year-round. In-depth analyses of the structural organization of communities from such habitats could have important implications for improving our understanding about factors promoting biodiversity and influencing robustness of species rich neotropical communities.

Intra-annual variation in network structure has been examined more extensively in subtropical and arctic communities while data on seasonal and temporal variation of tropical systems remain scarce [39, 69]. Nonetheless, a recent analysis of intra-annual variation in network structure for plant-pollinator communities in other seasonally dry habitats (Cerrado, Chaco, and the Pantanal) corroborates our findings that fewer plant-pollinator interactions occur during the dry season [70]. Even though differences in sampling and analytical approaches limit a direct comparison between our results, the study by Souza and colleagues, which was also carried in a *campo sujo* habitat of Cerrado, shows that all communities analyzed, including the Chaco and the Pantanal, had fewer interactions and a lower diversity of plants flowering during the dry season [70]. Furthermore, despite having a much lower diversity of bees than that observed for our study site, the plant-pollinator communities studied by Souza and colleagues were also characterized by the predominance of interactions involving social bees, especially honey bees, in both season [70]. These observations indicate that some generalities might be found among plant-pollinator communities from seasonally dry habitats and additional studies should help evaluate how seasonal and temporal variation in the structure of these communities can affect their stability over time.

A temporal analysis of a year-long active plant-pollinator community in the talar, a type of phytophysiognomy occurring in the Chaco biome of Argentina, showed that variation in network structural features (e.g., connectance) was associated with expansion and shrinking of the flowering/foraging bee communities during spring/summer versus autumn/winter months [63, 71]. Similar to the pattern observed in the Cerrado, high seasonal turnover in plant composition was paired with moderate turnover in pollinators which also connected the four seasonal interaction clusters observed in this subtropical habitat. Conversely, arctic communities, which are dominated by flies, have lower species richness (i.e., 17–31 plant and 26–76 pollinator species), a shorter period of activity (e.g., ~ 40–70 days/year) than tropical and subtropical systems, and undergo temporal change in interactions in proportionally less time [72, 73]. For instance, two temporal clusters corresponding to “early” and “late” season periods are recognized in an arctic island community from Greenland, which present distinct topologies despite being just nine days apart [72, 73]. Unlike the Cerrado community analyzed in our study, a few long-flowering “aseasonal” plants but not pollinator species appear to be the main connectors in these arctic plant-pollinator communities. Together, these studies and ours provide preliminary yet adequate evidence that despite many generalities shared between pollination and other mutualistic networks across latitudes [74, 75], some intra-annual variation in pollination networks appears to be system-specific and factors such as seasonality, community size, composition, and length of activity appear to influence the magnitude of change over time [70, 72, 73]. Therefore, targeted studies of temporal variation, inter- and intra-annually, of plant-pollinator communities from different habitats and climates are especially important not only to characterize these communities but also to estimate their resilience to future and expected changes in climate.

### Seasonality, community robustness, and biodiversity conservation

Our study focused on plant-pollinator interactions in a *campo sujo* area, a widespread type of Cerrado phytophysiognomy characterized by a relatively open vegetation mainly composed by grasses interleafed with herbaceous plants, and shrubs, with sparse occurrence of lianas and trees [16, 30]. While most of the Cerrado’s woody plants are able to cope with water stress by tapping on water reserves stored deep in the soil during the dry season, such water pockets remain hardly accessible for shallow-rooted herbs and shrubs, the predominant type of vegetation found in *campo sujo* [16, 33, 34, 37]. Therefore, germination and flowering during the dry season are more likely to be affected by water stress in *campo sujo*. Despite being a widespread vegetation type in this system, the Cerrado in its entire extension is known to be a mosaic of phytophysiognomies, which include open grasslands savannas without trees, shrub and herb dominated areas like the *campo sujo*, gallery forests, scrubland dominated by trees (Cerrado *sensu strictu*), and closed canopy vegetation (Cerradão) [16, 30, 76]. Further research should focus on evaluating the influence of seasonal drought on plant-pollinator communities from other Cerrado phytophysiognomies, especially those comprising a large percentage of woody species, as these could potentially function as source populations for those that become more vulnerable during the period of water stress.

Our simulations of species extinction events (i.e., species removal) indicated that removal of aseasonal, highly connected pollinator species resulted in a faster collapse of the networks than the removal of any other group of species comprised in the communities. In our models, rates of secondary extinctions were higher for pollinators upon plant removal than vice-versa, corroborating the principle that aseasonal pollinators represent key connecting elements for the whole community. This pattern is consistent with the changes in plant and pollinator degree (i.e., ecological generality) as well as connectance, among other properties, observed during the dry season.

Species loss in the community exerted a stronger impact on the patterns of secondary extinction (i.e., extinction based on lack of interactions partners) for the dry versus rainy season networks, these responses were not significantly different when we compared robustness between each seasonal and the overall network. Thus, our results show that cumulative accounts of interactions, irrespective of seasonality, yield a different and incorrect picture of network robustness. As already pointed out by others [39, 63, 70, 71, 75, 77], such cumulative estimates of species degree can lead to overestimation of partner redundancy as it assumes that in absence of one partner species, all other partners would be available at any given time of the year. For instance, most of the species that appear to have a large number of mutualistic partners in a cumulative analysis of the studied community, which includes interactions from both seasons, have most interactions concentrated in one season, remaining only weakly connected outside of their main primary season. In this context, our findings also highlight the importance of including information about floral rewards used by pollinators when evaluating community robustness, especially for communities with a large percentage of evolutionary specialized species [77]. For instance, Cerrado communities harbor a large diversity of oil-collecting bee species as well as oil-producing plants, some of which (from both trophic levels) ranked as top generalists in our network analyses based on species degree. However, these species are not true generalists given that plants that produce oils usually do not produce nectar and visitors are required to interact with many non-specialized floral hosts to acquire nectar which is equally essential to an oil bee as are oil and pollen. Conversely, plant species with oil-producing flowers lack nectar and thus rely on a restricted portion of the pollinator community, i.e., oil-collecting bees, for pollination. Hence, a superficial analysis of species degree, as those carried in studies considering cumulative accounts of interactions independent of the ecological significant of plant-pollinator interactions, would likely lead to an overestimation of partner redundancy for such species. Neotropical communities often comprise a large number of specialized plant-pollinator mutualisms, such as perfume producing flowers and perfume-collecting male orchid bees or resin-producing flowers and resin-collecting bees [12, 64]. Thus, we would expect that cumulative analyses of such communities would yield incorrect results.

While the link between climate change, especially in temperature, and phenological shifts in plants and animals has been well established in the Northern Hemisphere [78, 79], analyses evaluating such effects in tropical habitats are still scarce [5, 9, 80, 81]. Yet some studies already indicate that, in the Southern Hemisphere, precipitation, instead of temperature, may have a stronger effect on plant and animal phenology than temperature [5, 9, 80, 81]. Given the extent of areas covered by seasonally dry forests and savannas worldwide, their relevance for conservation of biodiversity, and the increasing need for baseline research to estimate effects of climate change for the persistence of natural communities a better understanding of plant-pollinator ecology from such habitats is much needed.

### Conclusions

Our results corroborate the idea that temporal and seasonal variation in species interactions are likely to influence network structural features relevant for understanding dynamics and robustness of plant-pollinator communities. The inclusion of such information might be especially important in the analyses of communities from highly seasonal habitats such as the seasonally dry savannas and forests from South America. Here, we have shown that the majority of plant-pollinator interactions occurs during either the dry or the rainy seasons and less than 3% of the interactions are observed in both periods. While strong seasonality in plant phenology drives high interaction turnover in this system while one third of the pollinator assemblage is aseasonal. Our findings indicate that the dry period that characterizes the Cerrado can lead to a reduction in flowering community size and this reduced availability of partners, most specifically floral hosts, can lead to a higher vulnerability to disturbance in this plant-pollinator community during this season. However, such change could be dependent upon the type of phytophysiognomy covering the area examined. Areas dominated by herbs and shrubs may be more vulnerable to disturbance during the dry season. The Cerrado is composed of a mosaic of phytophysiognomies, which include different proportions of plants with shallow root systems. Studies on plant-pollinator community, dynamics, resilience, and robustness need to consider vegetation cover. In the systems studied, aseasonal pollinators, comprising both specialized and social bees, function as keystone species and are especially important for the robustness of the overall system. We suggest that inclusion of resource use, in addition to seasonal information on species interactions, might be important and necessary for accurate analyses of ecology and robustness analyses of Neotropical communities that are subject to strong seasonality.

## Supporting information

S1 Fig Summary of climatic conditions at the study site in the period from 1980 to 2010. *Left figure*: Monthly precipitation in centimeters, stars indicate the average in millimeters and bars represent confidence intervals. *Right figure*: Monthly temperature in Celsius degrees, solid circles indicate the average high daytime temperatures, open circles show the average of the lowest nighttime values, and bars represent confidence intervals. Months on the horizontal axis reflect the seasonal periods defined in the analysis (see methods section). Red indicates dry season and blue rainy season months.
(JPG)

S2 Fig Rarefaction analyses of sampling effort for capturing interactions recorded during the dry season at the study site. (TIFF)

S3 Fig Rarefaction analyses of sampling effort for capturing interactions recorded during the rainy season at the study site. (TIFF)

S4 Fig Turnover in monthly bee-plant networks from Oct 2008 to Sep 2009. Every node in a monthly network represents a distinct plant or bee species. Each link between plant and bee nodes represent a unique pollination visit in the corresponding month. Links between monthly networks show interactions recorded in both monthly networks. Background of plot is color-coded to reflect the seasons: blue—rainy season; red—dry season. Lighter shaded areas demarcate the pollinator networks of each month, while darker areas demarcate the links present between monthly networks. Color of links represent the number of monthly networks in which the interaction was found. (Total no. of unique interactions: 434; No. of interactions present in 1–2 months: 414; 3 months: 18; 4 months: 2).
(PDF)

S5 Fig Trends between species turnover (*β*_S_) and interaction turnover (*β*_int_), interaction rewiring (*β*_rw_) and network dissimilarity due to *β*_S_ (*β*_st_). *β*_*S*_ has a strong and non-random positive correlation with *β*_int_ at the study site while *β*_st_ and *β*_rw_ do not associate with *β*_S_ (r_s_: Spearman’s correlation coefficient; *p*: *p*-values generated using Monte Carlo simulations).
(TIFF)

S1 Table Measures of dissimilarity in the plant-pollinator community between seasons. Dissimilarity measures were calculated using the respective elements and equations.
(DOCX)

S2 Table List of plant and pollinator species recorded from October 2008 to September 2009 at the study site, sorted by decreasing degree (i.e., highest to lowest number of interaction partners). In this table, *Degree* indicates the total number of different associations (or links) observed for each species of pollinator (Pol) or plant during the study year. *Cum*. = total counts for the entire observation period; *Rainy* = interactions recorded during the rainy season; *Dry* = interactions recorded during the dry season only; *A* = Aseasonal, indicating species which had an even distribution of interactions and were abundant in both seasons; *R* = Rainy, indicates species that were more abundant and established more than 2/3 of their interactions types (i.e., links) during the rainy season; *D* = Dry, indicates species that were more abundant and established more than 2/3 of their interactions types (i.e., links) during the dry season. Social bees indicated by *; solitary specialized oil-collecting species indicated by §.
(DOCX)

S3 Table List of plant and pollinator species recorded from October 2008 to September 2009 at the study site, sorted by interaction frequency (i.e., abundance of interactions observed). In this table, *Interaction Frequency* is a proxy for abundance and indicates how often each species was observed interacting with other species during each season. *Cum*. = total counts for the entire observation period; *Rainy* = interactions recorded during the rainy season; *Dry* = interactions recorded during the dry season only; *A* = Aseasonal, indicating species which had an even distribution of interactions and were abundant in both seasons; *R* = Rainy, indicates species that were more abundant and established more than 2/3 of their interactions types (i.e., links) during the rainy season; *D* = Dry, indicates species that were more abundant and established more than 2/3 of their interactions types (i.e., links) during the dry season. Social bees indicated by *; solitary specialized oil-collecting species indicated by §.
(DOCX)

S4 Table Degree distribution and statistics of the studied Cerrado plant-pollinator community. Degree distribution characteristics across seasons for the two communities quantified by fitting three alternative models: exponential, power-law, and truncated power law. The Akaike Information Criteria (AIC) values are shown for each fit. In all but one case (indicated with *), the truncated power law is the best fitting distribution.
(DOCX)

S5 Table Month-to-month turnover values for all dissimilarity measures calculated for the Cerrado plant-pollinator community studied. (DOCX)

S6 Table Correlation results for the relationships between turnover measures. r_s_: *Spearman*’s correlation coefficient;
*p*_s_: *p*-value of Spearman’s test;
*p*-value: generated using 10^5^ randomized networks for each month;
*β*_int_: interaction turnover;
*β*_rw_: interaction rewiring;
*β*_st_: interaction turnover due to species dissimilarity;
*β*_S_: species turnover.
(DOCX)

S7 Table Results of robustness analyses for the Cerrado community studied. The Area Under the Curve (AUC) values are shown for each curve (mean of 100 random removal sequences) followed by the proportion of nodes or resource service remaining after removal, and the values obtained for the 95% Confidence Intervals in parentheses. Pairs of dry and rainy season network robustness AUC values that are significantly different (95% CI’s of robustness curves do not overlap) are also flagged by an asterisk.
(DOCX)
