# Supplementary figures and images for: Seasonal variation of a plant-pollinator network in the Brazilian Cerrado: Implications for community structure and robustness
Source: PLoS One. 2019 Dec 2;14(12):e0224997. doi: 10.1371/journal.pone.0224997 (PMC6886790; doi:10.1371/journal.pone.0224997)

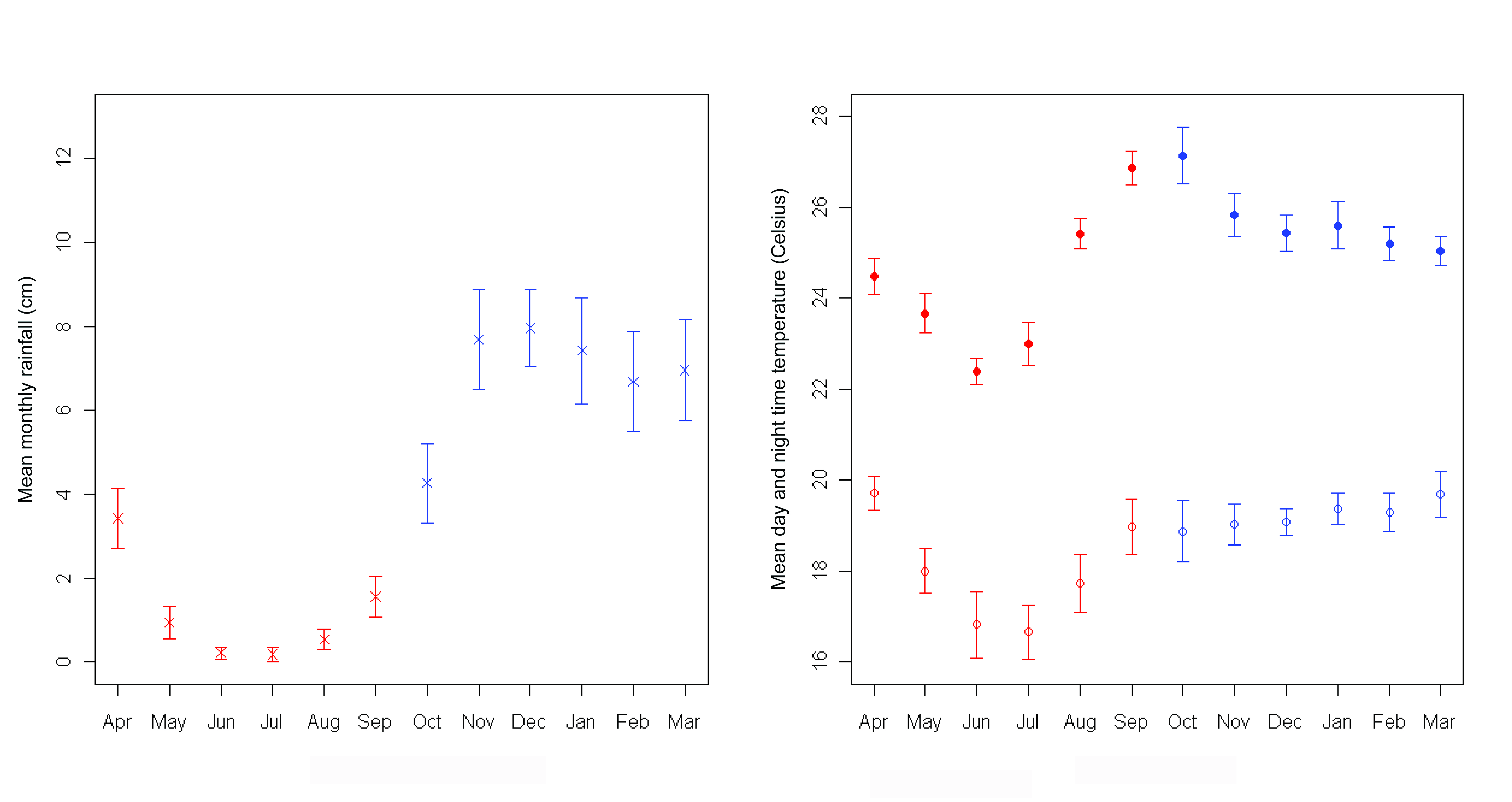

Supplement: S1 Fig — Left figure: Monthly precipitation in centimeters, stars indicate the average in millimeters and bars represent confidence intervals. Right figure: Monthly temperature in Celsius degrees, solid circles indicate the average high daytime temperatures, open circles show the average of the lowest nighttime values, and bars represent confidence intervals. Months on the horizontal axis reflect the seasonal periods defined in the analysis (see methods section). Red indicates dry season and blue rainy season months. (JPG) [file pone.0224997.s001.jpg]

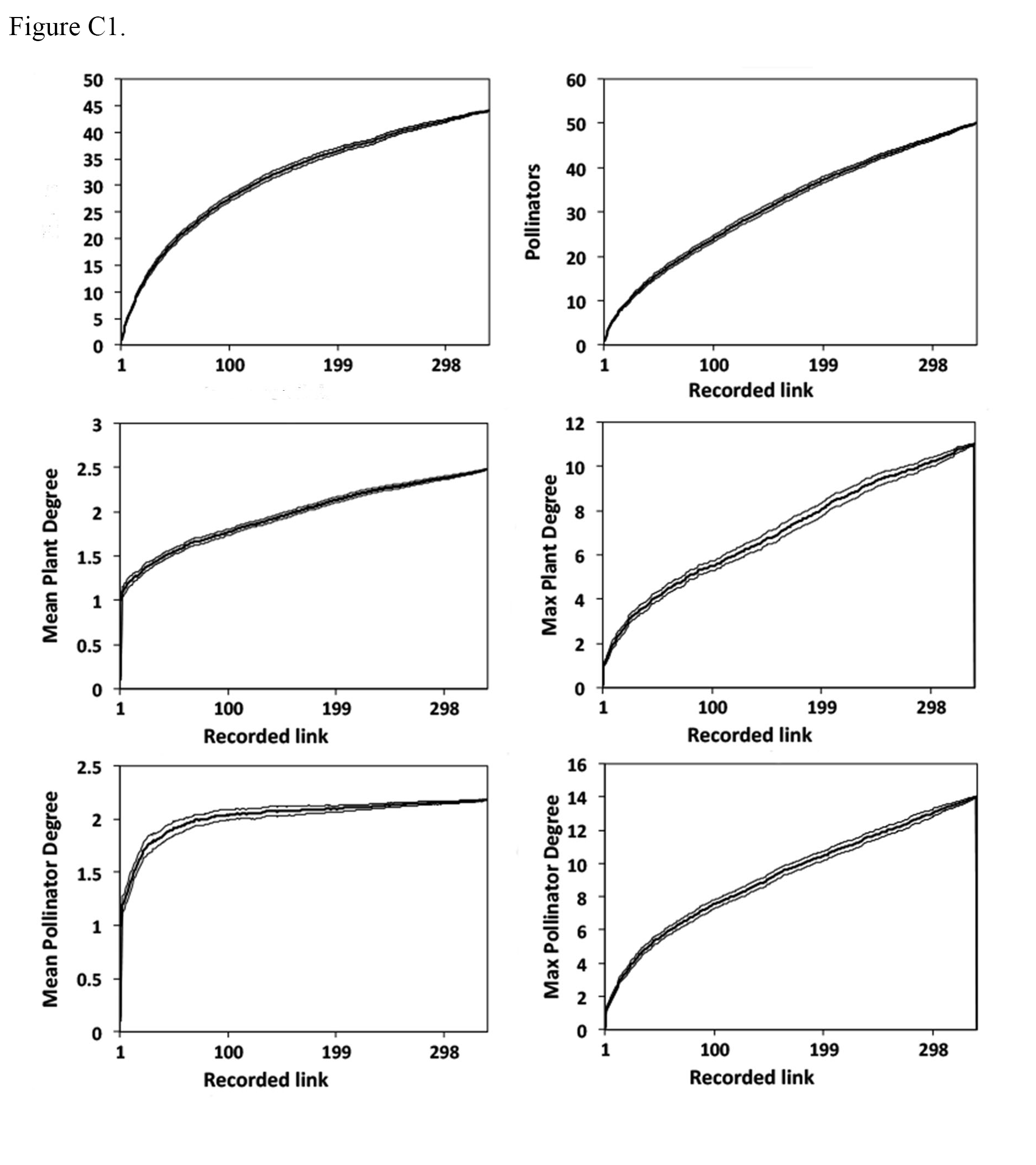

Supplement: S2 Fig — (TIFF) [file pone.0224997.s002.tiff]

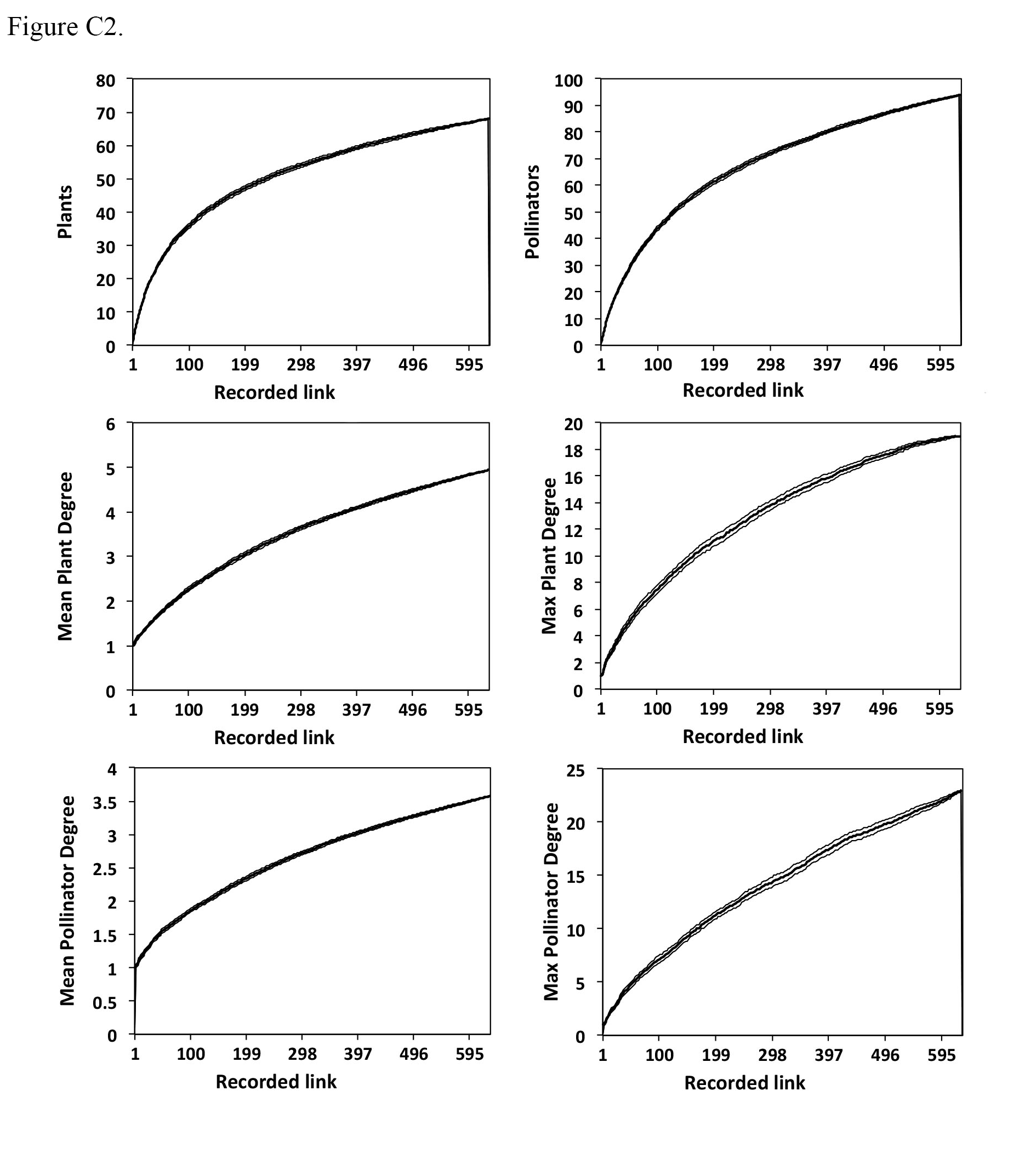

Supplement: S3 Fig — (TIFF) [file pone.0224997.s003.tiff]

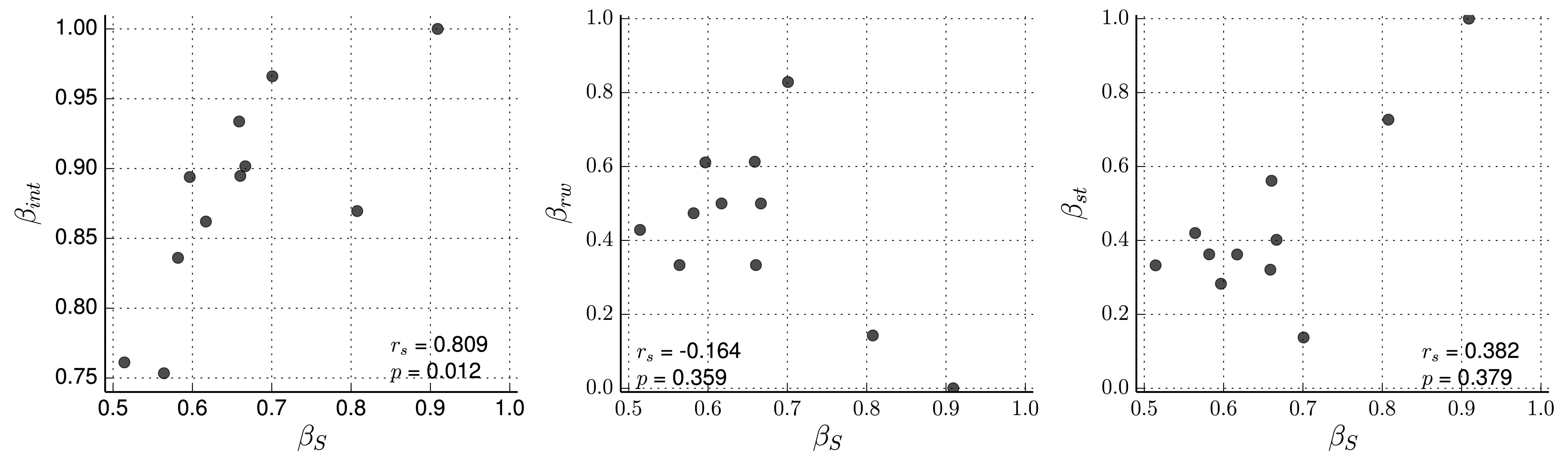

Supplement: S5 Fig — βS has a strong and non-random positive correlation with βint at the study site while βst and βrw do not associate with βS (rs: Spearman’s correlation coefficient; p: p-values generated using Monte Carlo simulations). (TIFF) [file pone.0224997.s005.tiff]
