# Supplementary material for: Seasonal variation of a plant-pollinator network in the Brazilian Cerrado: Implications for community structure and robustness
Source: PLoS One. 2019 Dec 2;14(12):e0224997. doi: 10.1371/journal.pone.0224997 (PMC6886790; doi:10.1371/journal.pone.0224997)
Supplement: S4 Fig — Every node in a monthly network represents a distinct plant or bee species. Each link between plant and bee nodes represent a unique pollination visit in the corresponding month. Links between monthly networks show interactions recorded in both monthly networks. Background of plot is color-coded to reflect the seasons: blue—rainy season; red—dry season. Lighter shaded areas demarcate the pollinator networks of each month, while darker areas demarcate the links present between monthly networks. Color of links represent the number of monthly networks in which the interaction was found. (Total no. of unique interactions: 434; No. of interactions present in 1–2 months: 414; 3 months: 18; 4 months: 2). (PDF) [file pone.0224997.s004.pdf]

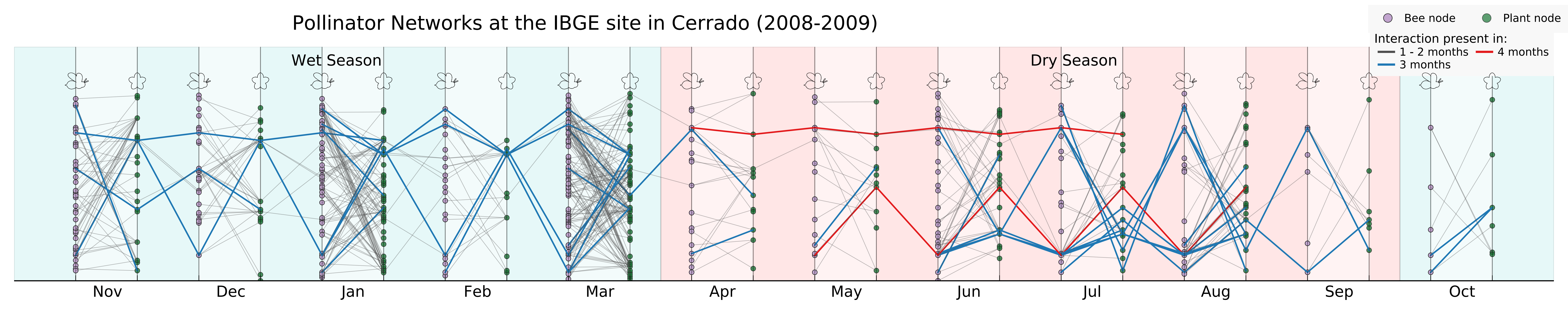
Pollinator Networks at the IBGE site in Cerrado (2008-2009)
Bee node
Plant node
Interaction present in:
1 - 2 months
3 months
4 months
Wet Season
Dry Season
Nov
Dec
Jan
Feb
Mar
Apr
May
Jun
Jul
Aug
Sep
Oct
