# Supplementary material for: Seasonal variation of a plant-pollinator network in the Brazilian Cerrado: Implications for community structure and robustness
Source: PLoS One. 2019 Dec 2;14(12):e0224997. doi: 10.1371/journal.pone.0224997 (PMC6886790; doi:10.1371/journal.pone.0224997)
Supplement: S1 Table — Dissimilarity measures were calculated using the respective elements and equations. (DOCX) [file pone.0224997.s006.docx]

**S1 Table.**

| **Measure** | **Definition** | **Elements** | **Reference** |
| --- | --- | --- | --- |
| *β*_int_ | Dissimilarity of interactions; Interaction turnover | All interactions | (50, 52) |
| *β*_rw_ | Dissimilarity of interactions between species present in both networks; Interaction rewiring | Interactions of shared species | (50, 52) |
| *β*_st_ | Dissimilarity of interactions due to species turnover |  | (50) |
| *β*_S_ | Dissimilarity in the species composition of both networks; Species turnover | Species names | (51) |
| *β*_po_ | Dissimilarity in the bee composition of both networks; bee turnover | Bee species name | This study |
| *β*_pl_ | Dissimilarity in the plant composition of both networks; plant turnover | Plant species name | This study |
