# Supplementary material for: Seasonal variation of a plant-pollinator network in the Brazilian Cerrado: Implications for community structure and robustness
Source: PLoS One. 2019 Dec 2;14(12):e0224997. doi: 10.1371/journal.pone.0224997 (PMC6886790; doi:10.1371/journal.pone.0224997)
Supplement: S3 Table — In this table, Interaction Frequency is a proxy for abundance and indicates how often each species was observed interacting with other species during each season. Cum. = total counts for the entire observation period; Rainy = interactions recorded during the rainy season; Dry = interactions recorded during the dry season only; A = Aseasonal, indicating species which had an even distribution of interactions and were abundant in both seasons; R = Rainy, indicates species that were more abundant and established more than 2/3 of their interactions types (i.e., links) during the rainy season; D = Dry, indicates species that were more abundant and established more than 2/3 of their interactions types (i.e., links) during the dry season. Social bees indicated by *; solitary specialized oil-collecting species indicated by §. (DOCX) [file pone.0224997.s008.docx]

**S3 Table.**

| **Node** | **Family** | **Species** | **Trophic level** | **Interaction frequency** | | | | | |
| --- | --- | --- | --- | --- | --- | --- | --- | --- | --- |
|  |  |  |  | **Cum.** | **Rainy** | **Dry** | **% Rainy** | **% Dry** | **Season** |
| 16 | Apidae | *Bombus brevivillus** | Pol | 86 | 40 | 46 | 0.47 | 0.53 | A |
| 91 | Apidae | *Paratrigona lineata** | Pol | 106 | 54 | 52 | 0.51 | 0.49 | A |
| 6 | Apidae | *Apis mellifera** | Pol | 154 | 45 | 109 | 0.29 | 0.71 | A |
| 17 | Apidae | *Bombus morio** | Pol | 43 | 17 | 26 | 0.40 | 0.60 | A |
| 102 | Apidae | *Trigona spinipes** | Pol | 60 | 30 | 30 | 0.50 | 0.50 | A |
| 90 | Apidae | *Paratetrapedia punctata*^§^ | Pol | 22 | 15 | 7 | 0.68 | 0.32 | A |
| 67 | Apidae | *Exomalopsis fulvofasciata* | Pol | 15 | 15 | 0 | 1.00 | 0.00 | R |
| 36 | Apidae | *Ceratina* (*Crewella*) sp. 2 | Pol | 12 | 9 | 3 | 0.75 | 0.25 | R |
| 37 | Apidae | *Ceratina* (*Crewella*) sp. 3 | Pol | 14 | 13 | 1 | 0.93 | 0.07 | R |
| 22 | Apidae | *Centris burgdorfi*^§^ | Pol | 13 | 6 | 7 | 0.46 | 0.54 | A |
| 56 | Apidae | *Epicharis iheringii*^§^ | Pol | 9 | 9 | 0 | 1.00 | 0.00 | R |
| 57 | Apidae | *Epicharis morio*^§^ | Pol | 21 | 18 | 3 | 0.86 | 0.14 | R |
| 66 | Apidae | *Exomalopsis campestris* | Pol | 11 | 10 | 1 | 0.91 | 0.09 | R |
| 100 | Apidae | *Tetrapedia diversipes*^§^ | Pol | 16 | 16 | 0 | 1.00 | 0.00 | R |
| 30 | Apidae | *Centris nitens*^§^ | Pol | 8 | 6 | 2 | 0.75 | 0.25 | R |
| 52 | Apidae | *Epicharis analis*^§^ | Pol | 15 | 15 | 0 | 1.00 | 0.00 | R |
| 53 | Apidae | *Epicharis bicolor*^§^ | Pol | 8 | 7 | 1 | 0.88 | 0.13 | R |
| 54 | Apidae | *Epicharis cockerelli*^§^ | Pol | 10 | 9 | 1 | 0.90 | 0.10 | R |
| 60 | Apidae | *Eufriesea violacens* | Pol | 7 | 7 | 0 | 1.00 | 0.00 | R |
| 73 | Apidae | *Geotrigona mombuca** | Pol | 21 | 19 | 2 | 0.90 | 0.10 | R |
| 82 | Megachilidae | *Megachile rubricate* | Pol | 8 | 7 | 1 | 0.88 | 0.13 | R |
| 96 | Apidae | *Scaptotrigona postica** | Pol | 49 | 48 | 1 | 0.98 | 0.02 | R |
| 108 | Apidae | *Xylocopa hirsutissima* | Pol | 7 | 7 | 0 | 1.00 | 0.00 | R |
| 18 | Apidae | *Bombus pauloensis** | Pol | 8 | 2 | 6 | 0.25 | 0.75 | D |
| 71 | Apidae | *Gaesischia belophora* | Pol | 8 | 5 | 3 | 0.63 | 0.38 | A |
| 87 | Apidae | *Monoeca mourei*^§^ | Pol | 10 | 10 | 0 | 1.00 | 0.00 | R |
| 99 | Apidae | *Tetragona quadrangula** | Pol | 11 | 5 | 6 | 0.45 | 0.55 | A |
| 101 | Apidae | *Tetrapedia peckoltii*^§^ | Pol | 10 | 9 | 1 | 0.90 | 0.10 | R |
| 104 | Apidae | *Tropidopedia flavolineata*^§^ | Pol | 7 | 5 | 2 | 0.71 | 0.29 | A |
| 31 | Apidae | *Centris varia*^§^ | Pol | 7 | 7 | 0 | 1.00 | 0.00 | R |
| 21 | Apidae | *Centris bicolor*^§^ | Pol | 4 | 2 | 2 | 0.50 | 0.50 | A |
| 35 | Apidae | *Ceratina* (*Crewella*) sp. 1 | Pol | 6 | 6 | 0 | 1.00 | 0.00 | R |
| 41 | Apidae | *Ceratina* (*Crewella*) sp. 7 | Pol | 4 | 3 | 1 | 0.75 | 0.25 | R |
| 51 | Megachilidae | *Epanthidium tigrinum* | Pol | 4 | 4 | 0 | 1.00 | 0.00 | R |
| 63 | Apidae | *Eulaema nigrita* | Pol | 6 | 6 | 0 | 1.00 | 0.00 | R |
| 65 | Apidae | *Exomalopsis auropilosa* | Pol | 5 | 1 | 4 | 0.20 | 0.80 | D |
| 69 | Apidae | *Exomalopsis* sp. 2 | Pol | 4 | 2 | 2 | 0.50 | 0.50 | A |
| 84 | Apidae | *Melissoptila richardia* | Pol | 5 | 3 | 2 | 0.60 | 0.40 | A |
| 5 | Megachilidae | *Anthodioctes megachiloides* | Pol | 3 | 2 | 1 | 0.67 | 0.33 | A |
| 9 | Halictidae | *Augochloropsis smithiana** | Pol | 3 | 1 | 2 | 0.33 | 0.67 | A |
| 11 | Halictidae | *Augochloropsis* sp. 2* | Pol | 3 | 3 | 0 | 1.00 | 0.00 | R |
| 28 | Apidae | *Centris scopipes*^§^ | Pol | 4 | 3 | 1 | 0.75 | 0.25 | R |
| 40 | Apidae | *Ceratina* (*Crewella*) sp. 6 | Pol | 8 | 8 | 0 | 1.00 | 0.00 | R |
| 55 | Apidae | *Epicharis flava*^§^ | Pol | 5 | 5 | 0 | 1.00 | 0.00 | R |
| 61 | Apidae | *Euglossa melanotricha* | Pol | 4 | 4 | 0 | 1.00 | 0.00 | R |
| 62 | Apidae | *Euglossa* sp. | Pol | 4 | 4 | 0 | 1.00 | 0.00 | R |
| 68 | Apidae | *Exomalopsis* sp. 1 | Pol | 3 | 3 | 0 | 1.00 | 0.00 | R |
| 80 | Megachilidae | *Megachile aureiventris* | Pol | 3 | 3 | 0 | 1.00 | 0.00 | R |
| 83 | Megachilidae | *Megachile terrestris* | Pol | 3 | 3 | 0 | 1.00 | 0.00 | R |
| 88 | Andrenidae | *Oxaea flavescens* | Pol | 9 | 9 | 0 | 1.00 | 0.00 | R |
| 109 | Apidae | *Xylocopa* sp. | Pol | 3 | 0 | 3 | 0.00 | 1.00 | D |
| 2 | Apidae | *Ancyloscelis* cft. *romeroi* | Pol | 4 | 4 | 0 | 1.00 | 0.00 | R |
| 4 | Megachilidae | *Anthidium sertanicola* | Pol | 2 | 2 | 0 | 1.00 | 0.00 | R |
| 12 | Halictidae | *Augochloropsis* sp. 3* | Pol | 2 | 0 | 2 | 0.00 | 1.00 | D |
| 13 | Halictidae | *Augochloropsis* sp. 4* | Pol | 2 | 1 | 1 | 0.50 | 0.50 | A |
| 14 | Halictidae | *Augochloropsis* sp. 5* | Pol | 2 | 2 | 0 | 1.00 | 0.00 | R |
| 29 | Apidae | *Centris tarsata*^§^ | Pol | 2 | 1 | 1 | 0.50 | 0.50 | A |
| 32 | Apidae | *Ceratalictus clonius** | Pol | 2 | 1 | 1 | 0.50 | 0.50 | A |
| 34 | Apidae | *Ceratina* (*Ceratinula*) sp. 2 | Pol | 2 | 1 | 1 | 0.50 | 0.50 | A |
| 38 | Apidae | *Ceratina* (*Crewella*) sp. 4 | Pol | 2 | 2 | 0 | 1.00 | 0.00 | R |
| 45 | Apidae | *Ctenioschelus goryi* | Pol | 2 | 1 | 1 | 0.50 | 0.50 | A |
| 75 | Megachilidae | *Larocanthidium* sp. | Pol | 2 | 1 | 1 | 0.50 | 0.50 | A |
| 47 | Halictidae | *Lasioglossum* (*Dialictus*) sp. 2* | Pol | 2 | 1 | 1 | 0.50 | 0.50 | A |
| 78 | Apidae | *Lophopedia pygmaea*^§^ | Pol | 2 | 2 | 0 | 1.00 | 0.00 | R |
| 93 | Apidae | *Partamona cupira** | Pol | 6 | 6 | 0 | 1.00 | 0.00 | R |
| 94 | Halictidae | *Pseudoagapostemon* sp.* | Pol | 2 | 2 | 0 | 1.00 | 0.00 | R |
| 95 | Halictidae | *Rhinocorynura* sp. | Pol | 2 | 2 | 0 | 1.00 | 0.00 | R |
| 110 | Apidae | *Xylocopa subcyanea* | Pol | 3 | 3 | 0 | 1.00 | 0.00 | R |
| 111 | Apidae | *Xylocopa vestita* | Pol | 2 | 0 | 2 | 0.00 | 1.00 | D |
| 1 | Halictidae | *Agapostemon chapadensis** | Pol | 1 | 0 | 1 | 0.00 | 1.00 | D |
| 3 | Apidae | *Ancyloscelis* sp. | Pol | 1 | 1 | 0 | 1.00 | 0.00 | R |
| 7 | Apidae | *Arhysoceble* sp. ^§^ | Pol | 1 | 1 | 0 | 1.00 | 0.00 | R |
| 8 | Halictidae | *Augochlorella ephyra** | Pol | 1 | 1 | 0 | 1.00 | 0.00 | R |
| 10 | Halictidae | *Augochloropsis* sp. 1* | Pol | 1 | 1 | 0 | 1.00 | 0.00 | R |
| 15 | Halictidae | *Augochloropsis* sp. 6* | Pol | 1 | 1 | 0 | 1.00 | 0.00 | R |
| 19 | Apidae | *Centris aenea*^§^ | Pol | 2 | 2 | 0 | 1.00 | 0.00 | R |
| 20 | Apidae | *Centris analis*^§^ | Pol | 1 | 1 | 0 | 1.00 | 0.00 | R |
| 23 | Apidae | *Centris fuscata*^§^ | Pol | 1 | 0 | 1 | 0.00 | 1.00 | D |
| 24 | Apidae | *Centris lateritia*^§^ | Pol | 1 | 0 | 1 | 0.00 | 1.00 | D |
| 25 | Apidae | *Centris machadoi*^§^ | Pol | 1 | 0 | 1 | 0.00 | 1.00 | D |
| 26 | Apidae | *Centris mocsaryi*^§^ | Pol | 2 | 2 | 0 | 1.00 | 0.00 | R |
| 27 | Apidae | *Centris rupestris*^§^ | Pol | 1 | 0 | 1 | 0.00 | 1.00 | D |
| 33 | Apidae | *Ceratina* (*Ceratinula*) sp. 1 | Pol | 1 | 1 | 0 | 1.00 | 0.00 | R |
| 39 | Apidae | *Ceratina* (*Crewella*) sp. 5 | Pol | 1 | 1 | 0 | 1.00 | 0.00 | R |
| 42 | Apidae | *Ceratina* (*Rhysoceratina*) sp. 1 | Pol | 2 | 2 | 0 | 1.00 | 0.00 | R |
| 43 | Megachilidae | *Coelioxys* sp. | Pol | 1 | 1 | 0 | 1.00 | 0.00 | R |
| 44 | Colletidae | *Colletes* sp. | Pol | 1 | 0 | 1 | 0.00 | 1.00 | D |
| 50 | Megachilidae | *Epanthidium aureocinctum* | Pol | 1 | 1 | 0 | 1.00 | 0.00 | R |
| 58 | Apidae | *Epicharis picta*^§^ | Pol | 1 | 1 | 0 | 1.00 | 0.00 | R |
| 59 | Apidae | *Epicharis xanthogastra*^§^ | Pol | 2 | 2 | 0 | 1.00 | 0.00 | R |
| 64 | Apidae | *Exaerete dentata* | Pol | 1 | 1 | 0 | 1.00 | 0.00 | R |
| 70 | Apidae | *Exomalopsis* sp. 3 | Pol | 1 | 0 | 1 | 0.00 | 1.00 | D |
| 72 | Apidae | *Gaesischia nigra* | Pol | 1 | 0 | 1 | 0.00 | 1.00 | D |
| 74 | Megachilidae | *Hypanthidium nigritulum* | Pol | 1 | 1 | 0 | 1.00 | 0.00 | R |
| 76 | Halictidae | *Lasioglossum* (*Dialictus*) sp. 1* | Pol | 1 | 0 | 1 | 0.00 | 1.00 | D |
| 46 | Halictidae | *Lasioglossum* (*D.*) *rostratum** | Pol | 1 | 1 | 0 | 1.00 | 0.00 | R |
| 48 | Halictidae | *Lasioglossum* (*D.*) sp. 3* | Pol | 1 | 1 | 0 | 1.00 | 0.00 | R |
| 49 | Halictidae | *Lasioglossum* (*D.*) sp. 4* | Pol | 2 | 0 | 2 | 0.00 | 1.00 | D |
| 77 | Megachilidae | *Lithurgus huberi* | Pol | 1 | 0 | 1 | 0.00 | 1.00 | D |
| 79 | Megachilidae | *Megachile* (*Pseudocentron*) sp. | Pol | 1 | 1 | 0 | 1.00 | 0.00 | R |
| 81 | Megachilidae | *Megachile frankieana* | Pol | 1 | 1 | 0 | 1.00 | 0.00 | R |
| 85 | Apidae | *Melissoptila* sp. | Pol | 1 | 0 | 1 | 0.00 | 1.00 | D |
| 86 | Apidae | *Monoeca pluricincta*^§^ | Pol | 2 | 0 | 2 | 0.00 | 1.00 | D |
| 89 | Apidae | *Paratetrapedia flaveola*^§^ | Pol | 2 | 0 | 2 | 0.00 | 1.00 | D |
| 92 | Halictidae | *Paraxystoglossa mimetica** | Pol | 7 | 7 | 0 | 1.00 | 0.00 | R |
| 97 | Apidae | *Schwarziana quadripunctata** | Pol | 2 | 0 | 2 | 0.00 | 1.00 | D |
| 98 | Halictidae | *Temnosoma* sp. | Pol | 1 | 1 | 0 | 1.00 | 0.00 | R |
| 103 | Apidae | *Tropidopedia carinata*^§^ | Pol | 1 | 1 | 0 | 1.00 | 0.00 | R |
| 105 | Apidae | *Xanthopedia larocai*^§^ | Pol | 1 | 1 | 0 | 1.00 | 0.00 | R |
| 106 | Apidae | *Xylocopa cearensis* | Pol | 1 | 0 | 1 | 0.00 | 1.00 | D |
| 107 | Apidae | *Xylocopa grisescens* | Pol | 1 | 1 | 0 | 1.00 | 0.00 | R |
|  |  |  |  |  |  |  |  |  |  |
| 147 | Asteraceae | *Dimerostemma vestitum* | P | 31 | 26 | 5 | 0.84 | 0.16 | R |
| 182 | Rubiaceae | *Palicourea coriacea* | P | 48 | 48 | 0 | 1.00 | 0.00 | R |
| 148 | Lythraceae | *Diplusodon oblongus* | P | 70 | 69 | 1 | 0.99 | 0.01 | R |
| 119 | Fabaceae | *Bauhinia dumosa* | P | 25 | 25 | 0 | 1.00 | 0.00 | R |
| 193 | Vochysiaceae | *Qualea parviflora* | P | 23 | 23 | 0 | 1.00 | 0.00 | R |
| 121 | Malpighiaceae | *Byrsonima basiloba* | P | 24 | 24 | 0 | 1.00 | 0.00 | R |
| 144 | Lythraceae | *Cuphea linarioides* | P | 19 | 19 | 0 | 1.00 | 0.00 | R |
| 153 | Fabaceae | *Galactia stereophylla* | P | 19 | 19 | 0 | 1.00 | 0.00 | R |
| 138 | Asteraceae | *Chresta sphaerocephala* | P | 18 | 0 | 18 | 0.00 | 1.00 | D |
| 166 | Asteraceae | *Lessingianthus brevipetiolatus* | P | 15 | 11 | 4 | 0.73 | 0.27 | A |
| 175 | Fabaceae | *Mimosa albolanata* | P | 55 | 47 | 8 | 0.85 | 0.15 | R |
| 165 | Asteraceae | *Lessingianthus bardanoides* | P | 13 | 8 | 5 | 0.62 | 0.38 | A |
| 117 | Malpighiaceae | *Banisteriopsis schizoptera* | P | 14 | 7 | 7 | 0.50 | 0.50 | A |
| 143 | Euphorbiaceae | *Croton goyazensis* | P | 87 | 7 | 80 | 0.08 | 0.92 | D |
| 116 | Malpighiaceae | *Banisteriopsis campestris* | P | 16 | 16 | 0 | 1.00 | 0.00 | R |
| 155 | Malpighiaceae | *Heteropterys campestris* | P | 13 | 12 | 1 | 0.92 | 0.08 | R |
| 159 | Lamiaceae | *Hyptis lythroides* | P | 32 | 0 | 32 | 0.00 | 1.00 | D |
| 178 | Fabaceae | *Mimosa radula* | P | 45 | 44 | 1 | 0.98 | 0.02 | R |
| 120 | Asteraceae | *Bidens graveolens* | P | 19 | 19 | 0 | 1.00 | 0.00 | R |
| 137 | Fabaceae | *Chamaecrista planaltoana* | P | 53 | 0 | 53 | 0.00 | 1.00 | D |
| 152 | Fabaceae | *Galactia heringeri* | P | 12 | 12 | 0 | 1.00 | 0.00 | R |
| 160 | Convolvulaceae | *Ipomoea contorquens* | P | 9 | 9 | 0 | 1.00 | 0.00 | R |
| 161 | Gentianaceae | *Irlbachia speciosa* | P | 10 | 2 | 8 | 0.20 | 0.80 | D |
| 164 | Asteraceae | *Lessingianthus argyrophyllus* | P | 15 | 12 | 3 | 0.80 | 0.20 | R |
| 168 | Asteraceae | *Lessingianthus ligulaefolius* | P | 20 | 16 | 4 | 0.80 | 0.20 | R |
| 131 | Fabaceae | *Centrosema bracteosum* | P | 9 | 9 | 0 | 1.00 | 0.00 | R |
| 142 | Fabaceae | *Crotalaria unifoliata* | P | 6 | 6 | 0 | 1.00 | 0.00 | R |
| 195 | Acanthaceae | *Ruellia incompta* | P | 6 | 1 | 5 | 0.17 | 0.83 | D |
| 205 | Velloziaceae | *Vellozia squamata* | P | 10 | 8 | 2 | 0.80 | 0.20 | R |
| 122 | Malpighiaceae | *Byrsonima coccolobifolia* | P | 5 | 5 | 0 | 1.00 | 0.00 | R |
| 149 | Lamiaceae | *Eriope complicata* | P | 13 | 9 | 4 | 0.69 | 0.31 | A |
| 176 | Fabaceae | *Mimosa gracilis* | P | 8 | 7 | 1 | 0.88 | 0.13 | R |
| 183 | Rubiaceae | *Palicourea rigida* | P | 8 | 8 | 0 | 1.00 | 0.00 | R |
| 196 | Rubiaceae | *Sabicea brasiliensis* | P | 6 | 5 | 1 | 0.83 | 0.17 | R |
| 124 | Malpighiaceae | *Byrsonima rigida* | P | 5 | 5 | 0 | 1.00 | 0.00 | R |
| 135 | Fabaceae | *Chamaecrista claussenii* | P | 5 | 2 | 3 | 0.40 | 0.60 | A |
| 154 | Rubiaceae | *Galianthe ramosa* | P | 10 | 10 | 0 | 1.00 | 0.00 | R |
| 115 | Asteraceae | *Aspilia foliacea* | P | 3 | 3 | 0 | 1.00 | 0.00 | R |
| 118 | Malpighiaceae | *Banisteriopsis stellaris* | P | 3 | 2 | 1 | 0.67 | 0.33 | A |
| 128 | Asteraceae | *Calea fruticosa* | P | 4 | 0 | 4 | 0.00 | 1.00 | D |
| 132 | Fabaceae | *Chamaecrista desvauxii* | P | 6 | 6 | 0 | 1.00 | 0.00 | R |
| 136 | Fabaceae | *Chamaecrista lundii* | P | 17 | 0 | 17 | 0.00 | 1.00 | D |
| 150 | Erythroxylaceae | *Erythroxylum campestre* | P | 14 | 0 | 14 | 0.00 | 1.00 | D |
| 157 | Lamiaceae | *Hypenia macrantha* | P | 3 | 1 | 2 | 0.33 | 0.67 | A |
| 162 | Acanthaceae | *Justicia picnophylla* | P | 5 | 5 | 0 | 1.00 | 0.00 | R |
| 180 | Ochnaceae | *Ouratea floribunda* | P | 5 | 0 | 5 | 0.00 | 1.00 | D |
| 185 | Malpighiaceae | *Peixotoa goiana* | P | 16 | 0 | 16 | 0.00 | 1.00 | D |
| 194 | Rubiaceae | *Richardia brasiliensis* | P | 5 | 0 | 5 | 0.00 | 1.00 | D |
| 199 | Verbenaceae | *Stachytarpheta chamissonis* | P | 3 | 1 | 2 | 0.33 | 0.67 | A |
| 203 | Melastomataceae | *Tibouchina candollea* | P | 3 | 3 | 0 | 1.00 | 0.00 | R |
| 113 | Lamiaceae | *Amasonia hirta* | P | 2 | 2 | 0 | 1.00 | 0.00 | R |
| 123 | Malpighiaceae | *Byrsonima pachyphylla* | P | 2 | 0 | 2 | 0.00 | 1.00 | D |
| 126 | Malpighiaceae | *Byrsonima verbascifolia* | P | 2 | 2 | 0 | 1.00 | 0.00 | R |
| 134 | Fabaceae | *Chamaecrista pohliana* | P | 2 | 2 | 0 | 1.00 | 0.00 | R |
| 158 | Lamiaceae | *Hyptis euneata* | P | 5 | 0 | 5 | 0.00 | 1.00 | D |
| 169 | Fabaceae | *Lupinus velutinus* | P | 6 | 3 | 3 | 0.50 | 0.50 | A |
| 172 | Convolvulaceae | *Merremia contorquens* | P | 2 | 2 | 0 | 1.00 | 0.00 | R |
| 177 | Fabaceae | *Mimosa lanuginosa* | P | 4 | 0 | 4 | 0.00 | 1.00 | D |
| 179 | Myrtaceae | *Myrcia* sp. | P | 2 | 2 | 0 | 1.00 | 0.00 | R |
| 187 | Malvaceae | *Peltaea obsita* | P | 2 | 2 | 0 | 1.00 | 0.00 | R |
| 190 | Melastomataceae | *Pterolepis repanda* | P | 2 | 2 | 0 | 1.00 | 0.00 | R |
| 192 | Vochysiaceae | *Qualea multiflora* | P | 2 | 2 | 0 | 1.00 | 0.00 | R |
| 202 | Malpighiaceae | *Tetrapterys ambigua* | P | 2 | 1 | 1 | 0.50 | 0.50 | A |
| 112 | Fabaceae | *Acosmium dasycarpum* | P | 1 | 1 | 0 | 1.00 | 0.00 | R |
| 114 | Bignoniaceae | *Arrabidhea sceptrum* | P | 1 | 1 | 0 | 1.00 | 0.00 | R |
| 125 | Malpighiaceae | *Byrsonima subterranea* | P | 1 | 1 | 0 | 1.00 | 0.00 | R |
| 127 | Malpighiaceae | *Byrsonima viminifolia* | P | 4 | 0 | 4 | 0.00 | 1.00 | D |
| 129 | Fabaceae | *Calliandra dysantha* | P | 3 | 0 | 3 | 0.00 | 1.00 | D |
| 130 | Malpighiaceae | *Camarea affinis* | P | 1 | 1 | 0 | 1.00 | 0.00 | R |
| 139 | Asteraceae | *Chromolaena leucocephala* | P | 2 | 0 | 2 | 0.00 | 1.00 | D |
| 141 | Connaraceae | *Connarus* sp. | P | 1 | 1 | 0 | 1.00 | 0.00 | R |
| 140 | Connaraceae | *Connarus suberosus* | P | 1 | 0 | 1 | 0.00 | 1.00 | D |
| 145 | Euphorbiaceae | *Dalechampia linearis* | P | 3 | 3 | 0 | 1.00 | 0.00 | R |
| 146 | Gentianaceae | *Deianira chiquitana* | P | 1 | 0 | 1 | 0.00 | 1.00 | D |
| 151 | Myrtaceae | *Eugenia complicata* | P | 1 | 0 | 1 | 0.00 | 1.00 | D |
| 156 | Asteraceae | *Hoehnephytum trixoides* | P | 1 | 1 | 0 | 1.00 | 0.00 | R |
| 163 | Asteraceae | *Lepidaploa aurea* | P | 1 | 0 | 1 | 0.00 | 1.00 | D |
| 167 | Asteraceae | *Lessingianthus durus* | P | 2 | 0 | 2 | 0.00 | 1.00 | D |
| 170 | Apocynaceae | *Macrosiphonia velame* | P | 1 | 1 | 0 | 1.00 | 0.00 | R |
| 171 | Apocynaceae | *Mandevilla novocapitalis* | P | 1 | 1 | 0 | 1.00 | 0.00 | R |
| 173 | Convolvulaceae | *Merremia tomentosa* | P | 1 | 0 | 1 | 0.00 | 1.00 | D |
| 174 | Melastomataceae | *Miconia albicans* | P | 1 | 1 | 0 | 1.00 | 0.00 | R |
| 181 | Ochnaceae | *Ouratea hexasperma* | P | 1 | 0 | 1 | 0.00 | 1.00 | D |
| 184 | Malvaceae | *Pavonia rosacampestris* | P | 1 | 1 | 0 | 1.00 | 0.00 | R |
| 186 | Malpighiaceae | *Peixotoa reticulata* | P | 1 | 0 | 1 | 0.00 | 1.00 | D |
| 188 | Burseraceae | *Protium ovatum* | P | 1 | 0 | 1 | 0.00 | 1.00 | D |
| 189 | Myrtaceae | *Psidium salutare* | P | 11 | 0 | 11 | 0.00 | 1.00 | D |
| 191 | Vochysiaceae | *Qualea grandiflora* | P | 1 | 1 | 0 | 1.00 | 0.00 | R |
| 197 | Solanaceae | *Solanum lycocarpum* | P | 2 | 1 | 1 | 0.50 | 0.50 | A |
| 198 | Solanaceae | *Solanum* sp. | P | 1 | 1 | 0 | 1.00 | 0.00 | R |
| 200 | Fabaceae | *Stylosanthes* sp. | P | 1 | 0 | 1 | 0.00 | 1.00 | D |
| 201 | Styracaceae | *Styrax ferrugineus* | P | 1 | 0 | 1 | 0.00 | 1.00 | D |
| 204 | Rubiaceae | *Tocoyena formosa* | P | 1 | 0 | 1 | 0.00 | 1.00 | D |
