# Supplementary material for: Seasonal variation of a plant-pollinator network in the Brazilian Cerrado: Implications for community structure and robustness
Source: PLoS One. 2019 Dec 2;14(12):e0224997. doi: 10.1371/journal.pone.0224997 (PMC6886790; doi:10.1371/journal.pone.0224997)
Supplement: S4 Table — Degree distribution characteristics across seasons for the two communities quantified by fitting three alternative models: exponential, power-law, and truncated power law. The Akaike Information Criteria (AIC) values are shown for each fit. In all but one case (indicated with *), the truncated power law is the best fitting distribution. (DOCX) [file pone.0224997.s009.docx]

**S4 Table.**

| Trophic Level | Degree | Degree Distribution  Fitting Model | Cumulative | Rainy | Dry |
| --- | --- | --- | --- | --- | --- |
| Plant | Mean |  | 4.7 | 4.8 | 2.5 |
|  | Maximum |  | 22 | 19 | 12 |
|  |  | Exponential fit | -63.2 | -58.2 | -40.6 |
|  |  | Power-law fit | -45.4 | -38.6 | -32.6 |
|  |  | Truncated power-law fit | -91.6 | -76.3 | -57.5 |
|  |  |  |  |  |  |
| Pollinators | Mean Degree |  | 3.9 | 3.5 | 2.3 |
|  | Maximum |  | 29 | 20 | 14 |
|  |  | Exponential fit | -63.5 | -58.6 | -27.5 |
|  |  | Power-law fit | -47.7 | -36.3 | -45.6* |
|  |  | Truncated power-law fit | -79.6 | -70.3 | -40.6 |
