# Supplementary material for: Seasonal variation of a plant-pollinator network in the Brazilian Cerrado: Implications for community structure and robustness
Source: PLoS One. 2019 Dec 2;14(12):e0224997. doi: 10.1371/journal.pone.0224997 (PMC6886790; doi:10.1371/journal.pone.0224997)
Supplement: S5 Table — (DOCX) [file pone.0224997.s010.docx]

**S5 Table.**

| **Year** | **Months** | ***β*_int_** | ***β*_rw_** | ***β*_st_** | ***β*_S_** | ***β*_po_** | ***β*_pl_** |
| --- | --- | --- | --- | --- | --- | --- | --- |
| 2008 | Oct-Nov | 1 | 0 | 1 | 0.909 | 0.86 | 1 |
| 2008 | Nov-Dec | 0.862 | 0.5 | 0.362 | 0.617 | 0.581 | 0.688 |
| 2008 | Dec-Jan | 0.894 | 0.611 | 0.283 | 0.597 | 0.543 | 0.673 |
| 2009 | Jan-Feb | 0.836 | 0.474 | 0.362 | 0.582 | 0.576 | 0.591 |
| 2009 | Feb-Mar | 0.934 | 0.613 | 0.321 | 0.659 | 0.636 | 0.692 |
| 2009 | Mar-Apr | 0.966 | 0.829 | 0.138 | 0.701 | 0.703 | 0.698 |
| 2009 | Apr-May | 0.895 | 0.333 | 0.561 | 0.66 | 0.613 | 0.727 |
| 2009 | May-Jun | 0.902 | 0.5 | 0.402 | 0.667 | 0.6 | 0.75 |
| 2009 | Jun-Jul | 0.753 | 0.333 | 0.42 | 0.564 | 0.707 | 0.405 |
| 2009 | Jul-Aug | 0.761 | 0.429 | 0.333 | 0.514 | 0.529 | 0.5 |
| 2009 | Aug-Sep | 0.87 | 0.143 | 0.727 | 0.808 | 0.76 | 0.852 |
