# Supplementary material for: Seasonal variation of a plant-pollinator network in the Brazilian Cerrado: Implications for community structure and robustness
Source: PLoS One. 2019 Dec 2;14(12):e0224997. doi: 10.1371/journal.pone.0224997 (PMC6886790; doi:10.1371/journal.pone.0224997)
Supplement: S6 Table — rs: Spearman’s correlation coefficient; ps: p-value of Spearman’s test; p-value: generated using 105 randomized networks for each month; βint: interaction turnover; βrw: interaction rewiring; βst: interaction turnover due to species dissimilarity; βS: species turnover. (DOCX) [file pone.0224997.s011.docx]

**S6 Table.**

| **Dissimilarity measures** | | **r_s_** | ***p*_s_** | ***p*-value** |
| --- | --- | --- | --- | --- |
| *β*_int_ | *β*_st_ | -0.027 | 0.937 | 0.037 |
| *β*_int_ | *β*_rw_ | 0.228 | 0.5 | 0.844 |
| *β*_int_ | *β*_S_ | 0.809 | 0.003 | 0.012 |
| *β*_int_ | *β*_po_ | 0.436 | 0.18 | 0.364 |
| *β*_int_ | *β*_pl_ | 0.773 | 0.005 | 0.012 |
| *β*_st_ | *β*_S_ | 0.382 | 0.247 | 0.379 |
| *β*_st_ | *β*_po_ | 0.555 | 0.077 | 0.158 |
| *β*_st_ | *β*_pl_ | 0.473 | 0.142 | 0.191 |
| *β*_S_ | *β*_po_ | 0.7 | 0.016 | 0.873 |
| *β*_S_ | *β*_pl_ | 0.964 | 0 | 0.002 |
| *β*_pl_ | *β*_po_ | 0.582 | 0.06 | 0.116 |
