# Supplementary material for: Seasonal variation of a plant-pollinator network in the Brazilian Cerrado: Implications for community structure and robustness
Source: PLoS One. 2019 Dec 2;14(12):e0224997. doi: 10.1371/journal.pone.0224997 (PMC6886790; doi:10.1371/journal.pone.0224997)
Supplement: S7 Table — The Area Under the Curve (AUC) values are shown for each curve (mean of 100 random removal sequences) followed by the proportion of nodes or resource service remaining after removal, and the values obtained for the 95% Confidence Intervals in parentheses. Pairs of dry and rainy season network robustness AUC values that are significantly different (95% CI’s of robustness curves do not overlap) are also flagged by an asterisk. (DOCX) [file pone.0224997.s012.docx]

**S7 Table.**

|  | **Cumulative** | **Rainy** | **Dry** |
| --- | --- | --- | --- |
| **Robustness to loss of pollinator species after plant removal** | | | |
| High to low degree | 0.48 (0.47±0.01) | 0.49* (0.51±0.01) | 0.43* (0.41±0.01) |
| Low to high degree | 0.76 (0.82±0.01) | 0.76* (0.83±0.01) | 0.67* (0.72±0.01) |
| Random | 0.67 (0.75±0.01) | 0.67* (0.75±0.01) | 0.57* (0.62±0.02) |
| **Robustness to loss of floral resources to pollinator after plant removal** | | | |
| High to low weighted degree | 0.38 (0.33±0.01) | 0.39* (0.35±0.01) | 0.35* (0.30±0.01) |
| Low to high weighted degree | 0.55 (0.56±0.01) | 0.55 (0.58±0.01) | 0.57 (0.60±0.01) |
| Random | 0.49 (0.50±0.01) | 0.49 (0.50±0.01) | 0.48 (0.49±0.01) |
| **Robustness to loss of plant species after pollinator removal** | | | |
| High to low degree | 0.50 (0.53±0.01) | 0.56* (0.61±0.01) | 0.44* (0.44±0.01) |
| Low to high degree | 0.84 (0.88±0.01) | 0.80* (0.85±0.01) | 0.74* (0.81±0.02) |
| Random | 0.70 (0.77±0.01) | 0.71* (0.79±0.01) | 0.63* (0.72±0.02) |
| **Robustness to loss of pollination services after pollinator removal** | | | |
| High to low weighted degree | 0.20 (0.13±0.001) | 0.27* (0.19±0.001) | 0.16* (0.1±0.001) |
| Low to high weighted degree | 0.62 (0.63±0.01) | 0.58* (0.60±0.01) | 0.67* (0.70±0.02) |
| Random | 0.49 (0.48±0.02) | 0.48* (0.49±0.01) | 0.51* (0.55±0.04) |
